# One-pot four-component reaction for convenient synthesis of functionalized 1-benzamidospiro[indoline-3,4'-pyridines]

**DOI:** 10.3762/bjoc.10.281

**Published:** 2014-11-14

**Authors:** Chao Wang, Yan-Hong Jiang, Chao-Guo Yan

**Affiliations:** 1 College of Chemistry & Chemical Engineering Yangzhou University, Yangzhou 225002, China

**Keywords:** acetylenedicarboxylate, benzohydrazide, 1,4-dihydropyridine, multicomponent reaction, one-pot reaction, spiro[indoline-3,4'-pyridine]

## Abstract

The one-pot four-component reaction of benzohydrazide (2-picolinohydrazide), acetylenedicarboxylate, isatins and malononitrile (ethyl cyanoacetate) with triethylamine as base catalyst afforded functionalized 1-benzamidospiro[indoline-3,4'-pyridines] in good yields. ^1^H NMR spectra indicated that an equilibrium of *cis/trans-*conformations exist in the obtained products.

## Introduction

The spirooxindole system is the core structure of many natural products and pharmaceutically important structures with notable structural complexity and biological activities of great interest [1–4]. Accordingly, many efficient and practical synthetic procedures have emerged for the synthesis of versatile spirooxindole-fused heterocycles [5–9]. In recent years, because of the emphasis on the development of green and sustainable chemistry, multicomponent reactions have been developed as efficient and potent tools for the preparation of structurally diverse molecules. Practically, multicomponent reactions based on the versatile reactivity of isatins and their 3-methylene derivatives have emerged in large numbers and become the new efficient protocols for the synthesis of various spirooxindoles [10–13].

On the other hand, the chemistry of Huisgen’s 1,4-dipoles, which are simply produced from the addition of nitrogen-containing nucleophiles to electron-deficient alkynes has aroused a lot of interest in organic synthesis due to its convenient formation and versatile reactivity [14–15]. The in situ formed Huisgen’s 1,4-dipoles were subsequently entrapped by various electrophiles and other reagents to finish a number of carbon–carbon bond formation reactions and heterocyclic construction processes [16–18]. A literature survey indicated that the common nitrogen-containing nucleophiles for generation of Huisgen’s 1,4-dipoles are aromatic heterocycles such as *N-*alkylimidazole, pyridine, quinoline, isoquinoline and primary aromatic amines. In recent years, other nitrogen-containing nucleophiles such as hydrazine and arylhydrazines are also used to generate Huisgen’s 1,4-dipoles in domino reactions [19–21]. Recently, we and Perumal have demonstrated that the four-component reaction of arylamine, acetylenedicarboxylate, isatin and malononitrile can afford the spiro[indoline-3,4’-pyridine] derivatives in satisfactory yields [22–24]. We envisioned that functionalized spiro[indoline-3,4’-pyridine] derivatives can be synthesized by employing other nitrogen-containing nucleophiles such as hydrazine and imines in the similar four-component reactions. In fact, the four-component reaction of hydrazine, acetylenedicarboxylate, isatin and malononitrile for the formation of spiro[indoline-3,4'-pyrano[2,3-c]pyrazoles] have been developed very recently by several groups [25–27]. Against this background and in continuation of our efforts toward the development of practical multicomponent reactions based on the reactivity of isatin and its derivatives [28–34], we herein wish to report the efficient synthesis of functionalized 1-benzamidospiro[indoline-3,4’-pyridines] via one-pot four-component reactions of benzohydrazide, acetylenedicarboxylate, isatin and malononitrile.

## Results and Discussion

According to the reaction conditions of the previously reported four-component reaction for the efficient synthesis of the functionalized spiro[indoline-3,4’-pyridine] derivatives [23] a mixture of benzohydrazide and dimethyl acetylenedicarboxylate in ethanol was firstly stirred at room temperature for about fifteen minutes. Then isatin and malononitrile as well as triethylamine as the base catalyst were introduced into reaction system. The subsequent reaction proceeded very smoothly at room temperature to give the 1'-benzamidospiro[indoline-3,4’-pyridines] **1a–d** in satisfactory yields (Table 1, entries 1–4). It is known that hydrazine reacts firstly with acetylenedicarboxylate to give the pyrazolone intermediate in the previously reported multicomponent reactions containing hydrazine and acetylenedicarboxylate [25–27]. Here, due to the protection of the benzoyl group, only the free amino group in benzohydrazide took part in the reaction to give the 1'-benzamido-substituted spiro[indoline-3,4’-pyridine]. It should be noted that pure products can be obtained by washing the formed precipitates from the reaction solution and no further purification process is needed. The similar reactions with ethyl cyanoacetate also produced the spiro compounds **1e–i** in 68–74% (Table 1, entries 5–9). The substituents on the isatins showed little effect on the yields. Further, 2-picolinohydrazide can also be utilized in the four-component reactions to give the corresponding 1'-picolinamidospiro[indoline-3,4’-pyridines] **1j–m** in satisfactory yields (Table 1, entries 10–13). These results indicate that this four-component reaction may have a widely variety of substrates.

**Table 1:** Synthesis of spiro[indoline-3,4'-pyridines] **1a–m** via four-component reaction.^a^

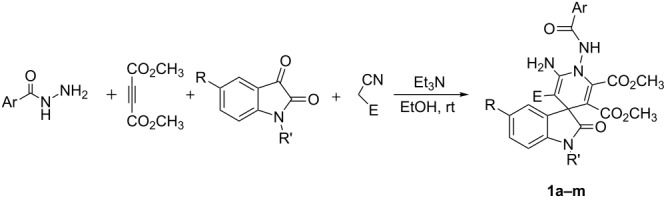						

Entry	Compd	Ar	R	R’	E	Yield (%, *cis/trans*)^b^

1	**1a**	C_6_H_5_	H	CH_2_C_6_H_4_	CN	72 (4:1)
2	**1b**	C_6_H_5_	F	CH_2_C_6_H_4_	CN	84 (6.5:1)
3	**1c**	*p-*CH_3_C_6_H_4_	H	H	CN	78
4	**1d**	*p-*CH_3_C_6_H_4_	F	CH_2_C_6_H_4_	CN	65 (6.5:1)
5	**1e**	C_6_H_5_	H	H	CO_2_Et	74 (5:1)
6	**1f**	C_6_H_5_	Cl	H	CO_2_Et	70 (5:1)
7	**1g**	C_6_H_5_	Cl	CH_2_C_6_H_4_	CO_2_Et	68 (6:1)
8	**1h**	*p-*CH_3_C_6_H_4_	Cl	H	CO_2_Et	69 (5:1)
9	**1i**	*p-*CH_3_C_6_H_4_	Cl	CH_2_C_6_H_4_	CO_2_Et	70 (6.5:1)
10	**1j**	2-C_5_H_4_N	CH_3_	H	CO_2_Et	80 (5:1)
11	**1k**	2-C_5_H_4_N	Cl	H	CO_2_Et	82 (4:1)
12	**1l**	2-C_5_H_4_N	Cl	CH_2_C_6_H_4_	CO_2_Et	68 (5:1)
13	**1m**	2-C_5_H_4_N	CH_3_	CH_2_C_6_H_4_	CO_2_Et	81 (3:1)

^a^Reaction conditions: arylhydrazide (1.0 mmol), acetylenedicarboxylate (1.0 mmol) in EtOH (15.0 mL), rt, 15 min; isatin (1.0 mmol), malononitrile or ethyl cyanoacetate (1.0 mmol), Et_3_N (0.2 mmol), rt, 24 h; ^b^Isolated yield.

**Figure 1:**
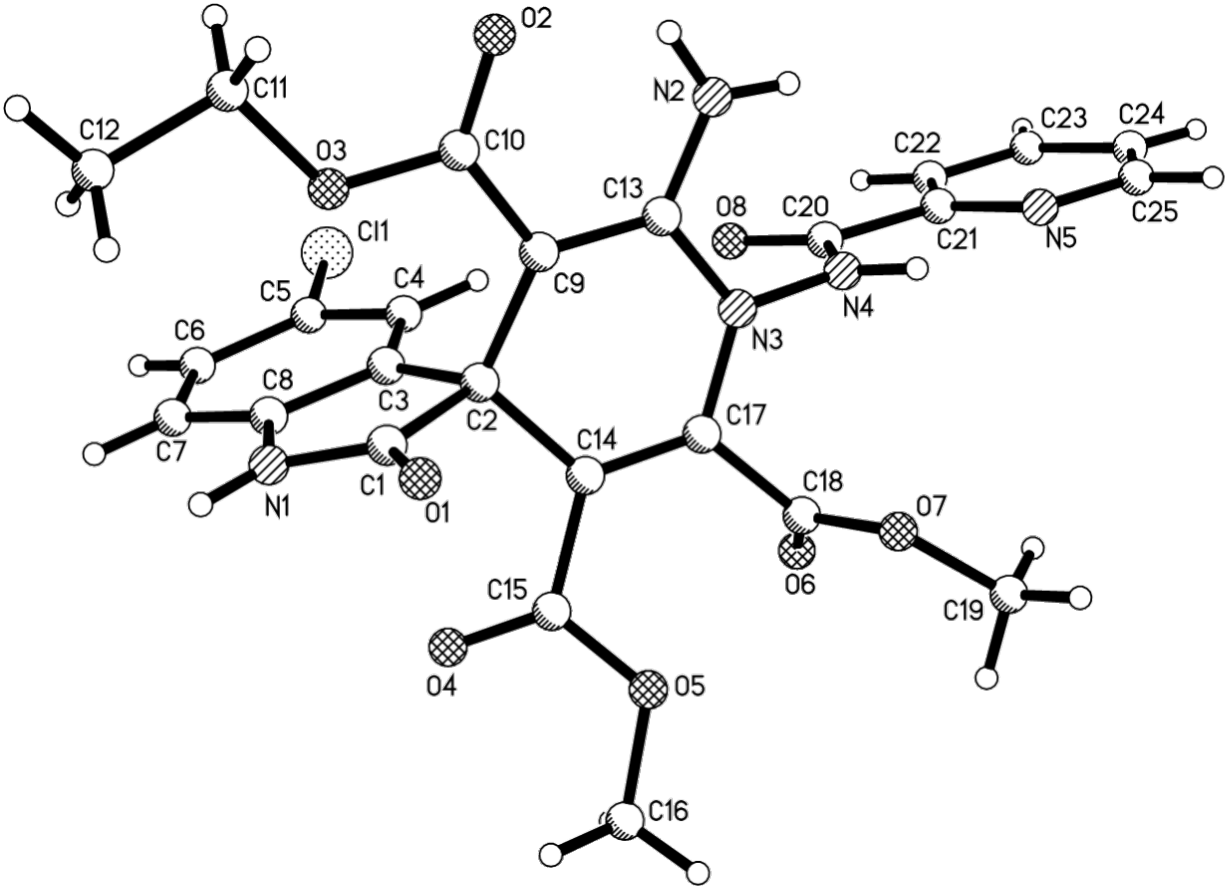
Molecular structure of spiro[indoline-3,4'-pyridine] **1k**.

**Scheme 1:**
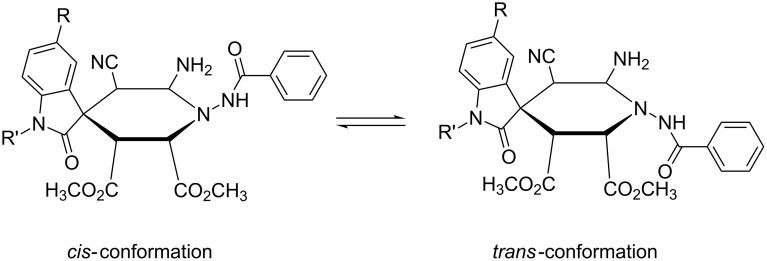
The dynamic equilibrium of *cis/trans*-conformation of spiro[indoline-3,4'-pyridine].

The structures of the prepared spiro[indoline-3,4’-pyridines] **1a–m** were fully characterized with IR, ^1^H, ^13^C NMR, HRMS spectra and were further confirmed by single-crystal X-ray diffraction determination of the compound **1k** (Figure 1). The ^1^H NMR spectra of compounds **1a–m** usually showed that two diastereoisomers with a ratio in range of 3:1 to 6.5:1 exist in the obtained products. But there is only one diastereoisomer in the product **1c** according to its ^1^H NMR spectrum. For an example, in the ^1^H NMR spectrum of compound **1a**, the amido group displays two singlets with a ratio of 4:1 at 11.46 and 11.38 ppm. The two singlets at 3.65 and 3.25 ppm are the signals of the two methoxy groups in the major isomer and the two singlets at 3.60 and 3.17 ppm are characteristic for the two methoxy groups in the minor isomer. From the molecular structure of spiro compound **1k** (Figure 1), it can be seen that the four carbon atoms and the nitrogen atom in the newly formed 1,4-dihydropyridyl ring exist nearly in one plane, while the C-4’ atom slightly deviates in this plane (0.318(4) Å). The phenyl group of the oxindole moiety and the 1'-picolinamido group exist in the same side of the 1,4-dihydropyridyl plane. By observing the crystal structure of spiro compound **1k**, we could conclude that the 1'-picolinamido group might exist in *cis*- or *trans*-position of the phenyl group of the oxindole moiety. Thus, the *cis/tran*s-conformations are in a dynamic equilibrium by inversion of the 1'-benzamido group (Scheme 1). The *cis*-conformation cannot be easily converted to the *trans*-conformation because the neighboring amino and methoxycarbonyl groups exhibit some steric hindrance for the inversion of the 1'-benzamido group. The ^1^H NMR spectra clearly indicated the existence of *cis/tran*s-conformations.

**Scheme 2:**
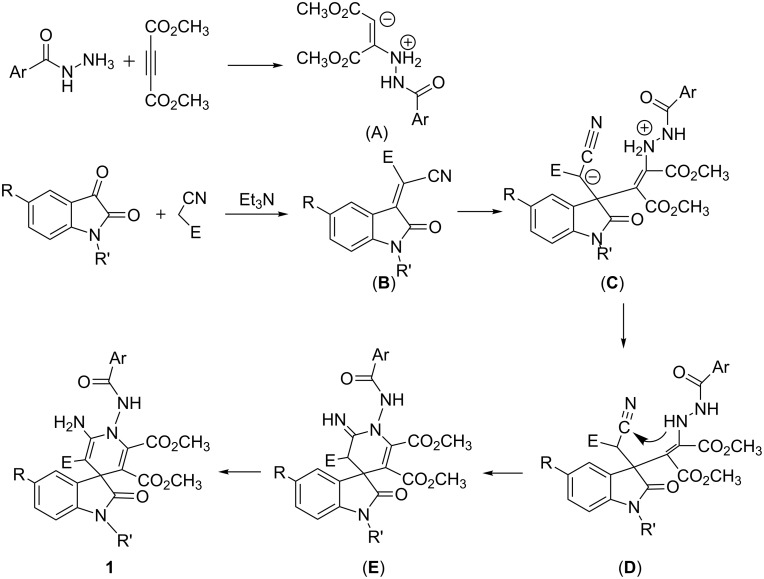
Proposed reaction mechanism for the four-component reaction.

In order to explain the formation of the spiro[indoline-3,4'-pyridines], a rational reaction mechanism is briefly proposed on the basis of similar reactions of Huigen’s 1,4-dipoles [22–24] (Scheme 2). Firstly, the addition of benzohydrazide to acetylenedicarboxylate results in an active zwitterionic intermediate (**A**). In the meantime, the condensation of isatin with malononitrile or ethyl cyanoacetate in the presence of triethylamine affords isatylidenemalononitrile or its derivative (**B**). Then the nucleophilic addition of the zwitterionic intermediate (**A**) to isatylidenemalononitrile (**B**) produces the adduct (**C**), which in turn transferres to intermediate (**D**) by immigration of a proton from the nitrogen atom to the carbon atom. Thirdly, the intramolecular reaction of the amino group with the cyano group gives a cyclized intermediate (**E**). Finally, the imino–enamino tautomerization results in the final spiro compound **1**. In this process, the initially formed zwitterrionic intermediate (**A**) does not cyclize to give pyrazolone intermediate as in the reaction of hydrazine with acetylenedicarboxylate. Thus, benzohydrazide shows very different reactivity to that of hydrazine in the four-component reaction.

## Conclusion

In summary, we have investigated the four-component reactions of benzohydrazide, acetylenedicarboxylate, isatins and malononitrile or ethyl cyanoacetate and successfully developed an efficient synthetic procedure for the preparation of functionalized 1-benzamidospiro[indoline-3,4'-pyridines]. Furthermore, the reaction mechanism and the dynamic equilibrium of *cis/trans*-conformations were also briefly discussed. This reaction provided new examples for the development of potential applications of Huisgen’s 1,4-dipoles in synthetic chemistry.

## Experimental

**Reagents and apparatus**: All reactions were monitored by TLC. Melting points were taken on a hot-plate microscope apparatus. IR spectra were obtained on a Bruker Tensor 27 spectrometer (KBr disc). ^1^H and ^13^C NMR spectra were recorded with a Bruker AV-600 spectrometer with DMSO-*d*_6_ as solvent and TMS as internal standard (600 and 150 MHz for ^1^H and ^13^C NMR spectra, respectively). HPLC/MS were measured at Bruker MicroTOF spectrometer. Single-crystal structure was determined on Bruker Smart-2 CCD diffractometer.

**General procedure for the synthesis of 1,4-dihydropyridines 1a–m via four-component reactions**: In a round bottom flask, a solution of benzohydrazide or 2-picolinohydrazide (1.0 mmol) and dimethyl acetylenedicarboxylate (1.0 mmol) in ethanol (15.0 mL) was stirred at room temperature for about fifteen minutes. Then, isatin (1.0 mmol), malononitrile or ethyl cyanoacetate (1.0 mmol) and triethylamine (0.2 mmol) was added. The mixture was stirred at room temperature for 24 hours. The resulting precipitates were collected by filtration and washed with cold alcohol to give the pure product for analysis.

**Dimethyl 2'-amino-1'-benzamido-1-benzyl-3'-cyano-2-oxo-1'*H*-spiro[indoline-3,4'-pyridine]-5',6'-dicarboxylate (1a)**: white solid, 72%; mp 222–224 °C; ^1^H NMR (600 MHz, DMSO-*d_6_*) δ *cis*-isomer: 11.46 (s, 1H, NH), 7.90–7.89 (m, 2H, ArH), 7.65–7.62 (m, 2H, ArH), 7.55 (brs, 2H, ArH), 7.50 (brs, 2H, ArH), 7.34 (brs, 2H, ArH), 7.29–7.28 (m, 1H, ArH), 7.21 (brs, 1H, ArH), 7.10 (d, *J* = 7.2 Hz, 1H, ArH), 6.82 (d, *J* = 7.2 Hz, 1H, ArH), 6.72 (brs, 2H, NH_2_), 4.98 (d, *J* = 15.0 Hz, 1H, CH_2_), 4.82 (d, *J* = 15.0Hz, 1H, CH_2_), 3.65 (s, 3H, OCH_3_), 3.25 (s, 3H, OCH_3_); *trans*-isomer: 11.38 (s, 1H, NH), 7.85–7.84 (m, 2H, ArH), 6.79 (brs, 2H, NH_2_), 3.60 (s, 3H, OCH_3_), 3.17 (s, 3H, OCH_3_); *cis/trans-*isomers: 4:1; ^13^C NMR (150 MHz, DMSO-*d_6_*) δ 177.3, 166.8, 163.6, 161.9, 152.1, 145.2, 141.3, 136.2, 135.2, 132.6, 131.1, 129.5, 128.6, 128.5, 128.4, 127.9, 127.6, 127.3, 124.1, 123.5, 122.8, 118.5, 108.8, 58.4, 52.9, 51.9, 49.4, 43.5; IR (KBr) υ: 3456, 2952, 2186, 1708, 1654, 1613, 1575, 1482, 1432, 1302, 1224, 1183, 1133, 1091, 1029, 936, 754, 697 cm^−1^; HRMS (ESI) (*m*/*z*): [M + Na]^+^ calcd. for C_31_H_25_N_5_NaO_6_: 586.1697; found: 586.1703.

## Supporting Information

Experimental details and detailed spectroscopic data of all new compounds are available as Supporting Information. Single crystal data for compounds **1k** (CCDC 1000773) has been deposited in the Cambridge Crystallographic Data Center.

File 1 Experimental details and spectroscopic data of all new compounds.
