# Supplementary material for: One-pot four-component reaction for convenient synthesis of functionalized 1-benzamidospiro[indoline-3,4'-pyridines]
Source: Beilstein J Org Chem. 2014 Nov 14;10:2671–6. doi: 10.3762/bjoc.10.281 (PMC4273213; doi:10.3762/bjoc.10.281)

Supporting Information

for

One-pot four-component reaction for convenient synthesis of functionalized 1-benzamidospiro[indoline-3,4'-pyridines]

Chao Wang, Yan-Hong Jiang and Chao-Guo Yan*

Address: College of Chemistry & Chemical Engineering Yangzhou University, Yangzhou 225002, China

Email: Chao-Guo Yan - cgyan@yzu.edu.cn

*Corresponding author

Experimental details and spectroscopic data of all new compounds

Characterization data of the new compounds **s2–s6**

^{1}H and ^{13}C NMR spectra **s7–s19**

General procedure for the synthesis of 1,4-dihydropyridines 1a-1m via four-component reactions: In a round bottom flask, a solution of benzohydrazide or 2-picolinohydrazide (1.0 mmol) and dimethyl acetylenedicarboxylate (1.0 mmol) in ethanol (15.0 mL) was stirred at room temperature for about fifteen minutes. Then, isatin (1.0 mmol), malononitrile or ethyl cyanoacetate (1.0 mmol) and triethylamine (0.2 mmol) was added. The mixture was stirred at room temperature for 24 hours. The resulting precipitates were collected by filtration and washed with cold alcohol to give the pure product for analysis.

Dimethyl 2'-amino-1'-benzamido-1-benzyl-3'-cyano-2-oxo-1'*H*-spiro[indoline-3,4'-pyridine]-5',6'-dicarboxylate (1a): white solid, 72%, m.p. 222~224℃; ^{1}H NMR (600 MHz, DMSO-d_6) δ: *cis*-isomer: 11.46 (s, 1H, NH), 7.90~7.89 (m, 2H, ArH), 7.65~7.62 (m, 2H, ArH), 7.55 (brs, 2H, ArH), 7.50 (brs, 2H, ArH), 7.34 (brs, 2H, ArH), 7.29~7.28 (m, 1H, ArH), 7.21 (brs, 1H, ArH), 7.10 (d, J = 7.2Hz, 1H, ArH), 6.82 (d, J = 7.2Hz, 1H, ArH), 6.72 (brs, 2H, NH_2), 4.98 (d, J = 15.0Hz, 1H, CH_2), 4.82 (d, J = 15.0Hz, 1H, CH_2), 3.65 (s, 3H, OCH_3), 3.25 (s, 3H, OCH_3); *trans*-isomer: 11.38 (s, 1H, NH), 7.85~7.84 (m, 2H, ArH), 6.79 (brs, 2H, NH_2), 3.60 (s, 3H, OCH_3), 3.17 (s, 3H, OCH_3). *cis/trnas* isomers: 4:1. ^{13}C NMR (150 MHz, DMSO-d_6) δ: 177.3, 166.8, 163.6, 161.9, 152.1, 145.2, 141.3, 136.2, 135.2, 132.6, 131.1, 129.5, 128.6, 128.5, 128.4, 127.9, 127.6, 127.3, 124.1, 123.5, 122.8, 118.5, 108.8, 58.4, 52.9, 51.9, 49.4, 43.5; IR (KBr) υ: 3456, 2952, 2186, 1708, 1654, 1613, 1575, 1482, 1432, 1302, 1224, 1183, 1133, 1091, 1029, 936, 754, 697 cm^{-1}; MS (*m/z*): HRMS (ESI) Calcd. for $C_{31}H_{25}N_5NaO_6$ ([M+Na]$^+$): 586.1697. Found: 586.1703.

Dimethyl 2'-amino-1'-benzamido-1-benzyl-3'-cyano-5-fluoro-2-oxo-1'*H*-spiro[indoline-3,4'-pyridine]-5',6'-dicarboxylate (1b): white solid, 84%, m.p. 222~224℃; ^{1}H NMR (600 MHz, DMSO-d_6) δ: *cis*-isomer: 11.50 (s, 1H, NH), 7.90 (d, J = 7.2Hz, 2H, ArH), 7.67~7.62 (m, 1H, ArH), 7.56 (t, J = 7.2Hz, 2H, ArH), 7.51~7.48 (m, 3H, ArH), 7.35~7.33 (m, 2H, ArH), 7.29 (d, J = 7.2Hz, 1H, ArH), 7.07 (t, J = 8.4Hz, 1H, ArH), 6.84 (brs, 1H, ArH), 6.78 (s, 2H, NH_2), 4.99 (d, J = 15.6Hz, 1H, CH_2), 4.82 (d, J = 15.6Hz, 1H, CH_2), 3.67 (s, 3H, OCH_3), 3.29 (s, 3H, OCH_3); *trans*-isomer: 11.30 (s, 1H, NH), 7.83 (d, J = 7.2Hz, 2H, ArH), 6.82 (s, 2H, NH_2), 3.61 (s, 3H, OCH_3), 3.22 (s, 3H, OCH_3). *cis/trans* isomers: 6.5:1. ^{13}C NMR (150 MHz, DMSO-d_6) δ: 177.1, 167.1, 163.5, 161.8, 159.6, 158.0, 152.4, 145.3, 137.5, 136.8, 136.0, 132.7, 130.9, 128.5, 128.4, 128.0, 127.6, 127.4, 118.3, 115.1, 114.9, 111.8, 111.7, 110.0, 109.9, 103.1, 58.0, 53.0, 52.0, 49.8, 43.6; IR (KBr) υ: 3456, 2952, 2186, 1708, 1654, 1613, 1575, 1482, 1432, 1302, 1224, 1183, 1133, 1091, 1029, 936, 754, 697 cm^{-1}; MS (*m/z*): HRMS (ESI) Calcd. for $C_{31}H_{24}FN_5NaO_6$ ([M+Na]$^+$): 604.1603. Found: 604.1597.

Dimethyl 2'-amino-3'-cyano-1'-(4-methylbenzamido)-2-oxo-1'*H*-spiro[indoline-3,4'-pyridine]-5',6'-dicarboxylate (1c): white solid, 78%, m.p. 222~224℃; ^{1}H NMR (600 MHz, DMSO-d_6) δ: 11.29 (s, 1H, NH), 10.43 (brs, 1H, NH), 7.79 (d, J = 6.0Hz, 2H, ArH), 7.56 (d, J = 6.0Hz, 1H, ArH), 7.35 (d, J = 6.0Hz, 2H, ArH), 7.19 (brs, 1H, ArH), 6.80 (d, J = 7.2Hz, 1H, ArH), 6.56 (brs, 2H, NH_2), 3.63 (s, 3H, OCH_3), 3.39 (s, 3H, OCH_3), 2.39 (s, 3H, CH_3); ^{13}C NMR (150 MHz, DMSO-d_6) δ: 178.8, 166.7, 163.6, 162.0, 156.8, 152.0, 144.9, 143.5, 142.8, 140.7, 136.1, 129.0, 128.5, 128.2, 127.9, 124.3, 121.9, 118.5, 109.1, 103.4, 58.8, 52.9, 51.8, 49.7, 21.1; IR (KBr) υ: 3436, 3357, 3318, 2953, 2194, 1743, 1695, 1660, 1616, 1578, 1478, 1429, 1329, 1304, 1266, 1236, 1185, 1133, 1095, 1042, 977, 922, 896, 831, 755 cm^{-1}; MS (*m/z*): HRMS (ESI) Calcd. for $C_{25}H_{21}N_5NaO_6$ ([M+Na]$^+$): 510.1384. Found: 510.1381.

Dimethyl 2'-amino-1-benzyl-3'-cyano-5-fluoro-1'-(4-methylbenzamido)-2-oxo-1'*H*-spiro-[indoline-3,4'-pyridine]-5',6'-dicarboxylate (1d): white solid, 65%, m.p. 237~239℃; ^{1}H NMR (600 MHz, DMSO-d_6) δ: *cis*-isomer: 11.43 (s, 1H, NH), 7.81 (d, *J* = 6.0Hz, 2H, ArH), 7.51~7.48 (m, 3H, ArH), 7.35~7.34 (m, 4H, ArH), 7.29~7.28 (m, 1H, ArH), 7.08 (s, 1H, ArH), 6.83 (s, 2H, NH_2), 6.79 (s, 1H, ArH), 4.99 (d, *J* = 15.0Hz, 1H, CH_2), 4.82 (d, *J* = 15.0Hz, 1H, CH_2), 3.65 (s, 3H, OCH_3), 3.29 (s, 3H, OCH_3), 2.39 (s, 3H, CH_3); *cis/trans* isomers: 11.23 (s, 1H, NH), 7.74 (d, *J* = 6.0Hz, 2H, ArH), 6.93 (s, 2H, NH_2), 3.59 (s, 3H, OCH_3), 3.21 (s, 3H, OCH_3). *cis/trans* isomers: 6.5:1. ^{13}C NMR (150 MHz, DMSO-d_6) δ: 177.1, 167.0, 163.4, 161.8, 160.0, 158.0, 152.4, 145.4, 143.0, 137.4, 136.8, 136.7, 136.0, 129.1, 128.5, 128.1, 128.0, 127.6, 127.4, 118.3, 115.1, 114.9, 111.9, 111.7, 110.0, 109.9, 103.0, 58.0, 53.0, 52.0, 49.8, 43.6, 21.1; IR (KBr) υ: 3466, 3292, 2953, 2185, 1754, 1716, 1652, 1617, 1570, 1528, 1491, 1432, 1325, 1295, 1265, 1228, 1175, 1128, 1090, 1028, 938, 876, 822, 748 cm^{-1}; MS (*m*/*z*): HRMS (ESI) Calcd. for $C_{32}H_{26}FN_5NaO_6$ ($[M+Na]^+$): 6181.759. Found: 618.1752.

3'-Ethyl 5',6'-dimethyl 2'-amino-1'-benzamido-2-oxo-1'*H*-spiro[indoline-3,4'-pyridine]-3',5',6'-tricarboxylate (1e): white solid, 74%, m.p. 212~214℃; ^{1}H NMR (600 MHz, DMSO-d_6) δ: *cis*-isomers: 11.46 (s, 1H, NH), 10.14 (s, 1H, NH), 7.93~7.89 (m, 2H, ArH), 7.78 (brs, 2H, NH_2), 7.66~7.64 (m, 1H, ArH), 7.56 (t, *J* = 7.8Hz, 2H, ArH), 7.49 (d, *J* = 7.2Hz, 1H, ArH), 7.11~7.09 (m, 1H, ArH), 6.91~6.88 (m, 1H, ArH), 6.68 (d, *J* = 7.2Hz, 1H, ArH), 3.77~3.69 (m, 2H, OCH_2), 3.63 (s, 3H, OCH_3), 3.37 (s, 3H, OCH_3), 0.85 (t, *J* = 7.2Hz, 3H, CH_3); *trans*-isomer. 11.37 (s, 1H, NH), 7.97 (brs, 2H, NH_2), 3.57 (s, 3H, OCH_3), 3.31 (s, 3H, OCH_3). *cis/trans* isomers: 5:1. ^{13}C NMR (150 MHz, DMSO-d_6) δ: 180.1, 167.9, 166.6, 163.8, 162.4, 153.0, 152.9, 142.9, 142.6, 137.0, 132.6, 131.2, 128.5, 127.9, 127.7, 124.0, 120.9, 108.0, 106.7, 77.7, 61.9, 58.7, 52.7, 51.5, 51.3, 49.9, 45.8, 13.1; IR (KBr) υ: 3393, 3239, 2984, 1720, 1699, 1652, 1616, 1482, 1434, 1396, 1375, 1328, 1299, 1247, 1200, 1135, 1097, 1071, 1053, 1027, 934, 882, 848, 760 cm^{-1}; MS (*m*/*z*): HRMS (ESI) Calcd. for $C_{26}H_{25}N_4O_8$ ($[M+H]^+$): 521.1667. Found: 521.1665.

3'-Ethyl 5',6'-dimethyl 2'-amino-1'-benzamido-5-chloro-2-oxo-1'*H*-spiro[indoline-3,4'-pyridine]-3',5',6'-tricarboxylate (1f): yellow solid, 70%, m.p. 214~216℃; ^{1}H NMR (600 MHz, DMSO-d_6) δ: *cis*-isomer: 11.50 (s, 1H, NH), 10.30 (s, 1H, NH), 7.92 (d, *J* = 7.2Hz, 2H, ArH), 7.80 (brs, 2H, NH_2), 7.67~7.64 (m, 1H, ArH), 7.58~7.55 (m, 2H, ArH), 7.51 (s, 1H, ArH), 7.18~7.16 (m, 1H, ArH), 6.71 (d, *J* = 7.8Hz, 1H, ArH), 3.81~3.73 (m, 2H, OCH_2), 3.65 (s, 3H, OCH_3), 3.41 (s, 3H, OCH_3), 0.88 (t, *J* = 7.2Hz, 3H, CH_3); *cis*-isomer: 11.30 (s, 1H, NH), 7.89 (d, *J* = 7.2Hz, 2H, ArH), 7.79 (brs, 2H, NH_2), 3.58 (s, 3H, OCH_3), 3.36 (s, 3H, OCH_3). *cis/trans* isomer: 5:1.^{13}C NMR (150 MHz, DMSO-d_6) δ: 180.5, 167.6, 166.9, 163.6, 162.3, 153.0, 143.2, 141.6, 138.9, 132.7, 131.0, 128.6, 127.9, 127.6, 124.7, 124.0, 109.5, 106.3, 77.4, 62.0, 58.9, 52.9, 51.7, 51.5, 50.1, 13.1; IR (KBr) υ: 3403, 3201, 2954, 1713, 1655, 1614, 1480, 1436, 1374, 1300, 1250, 1216, 1177, 1139, 1104, 1027, 938, 886, 816, 777 cm^{-1}; MS (*m*/*z*): HRMS (ESI) Calcd. for $C_{26}H_{24}ClN_4O_8$ ($[M+H]^+$): 555.1277. Found: 555.1276.

3'-Ethyl 5',6'-dimethyl 2'-amino-1'-benzamido-1-benzyl-5-chloro-2-oxo-1'*H*-spiro[indoline-3,4'-pyridine]-3',5',6'-tricarboxylate (1g): white solid, 68%, m.p. 216~218℃; ^{1}H NMR (600 MHz, DMSO-d_6) δ: *cis*-isomer: 11.58 (s, 1H, NH), 8.10~7.85 (m, 4H, NH_2, ArH), 7.67~7.63 (m,

1H, ArH), 7.58~7.54 (m, 4H, ArH), 7.52 (s, 1H, ArH), 7.38~7.35 (m, 2H, ArH), 7.30~7.28 (m, 1H, ArH), 7.25 (d, J = 7.2Hz, 1H, ArH), 6.99 (d, J = 8.4Hz, 1H, ArH), 4.91 (d, J = 15.6Hz, 1H, CH_2), 4.72 (d, J = 15.6Hz, 1H, CH_2), 3.83~3.77 (m, 1H, OCH_2), 3.64 (s, 3H, OCH_3), 3.45~3.40 (m, 1H, OCH_2), 3.13 (s, 3H, OCH_3), 0.62 (t, J = 7.2Hz, 3H, CH_3); *trans*-isomer: 11.36 (s, 1H, NH), 3.58 (s, 3H, OCH_3), 3.04 (s, 3H, OCH_3), 0.55 (t, J = 7.2Hz, 3H, CH_3). *cis/trans* isomers: 6:1. ^{13}C NMR (150 MHz, DMSO-d_6) δ: 178.9, 167.4, 166.9, 163.7, 162.1, 153.2, 143.6, 142.4, 138.3, 136.6, 132.7, 131.0, 128.6, 12.5, 128.4, 127.9, 127.6, 127.4, 125.8, 123.8, 109.0, 105.7, 77.1, 58.6, 52.9, 51.6, 49.5, 44.2, 13.7; IR (KBr) υ: 3372, 2948, 1749, 1720, 1691, 1663, 1607, 1478, 1433, 1369, 1331, 1294, 1249, 1204, 1175, 1141, 1110, 1026, 940, 887, 815, 780, 741 cm^{-1}; MS (*m*/*z*): HRMS (ESI) Calcd. for $C_{33}H_{29}ClN_4NaO_8$ ([M+Na]$^+$): 667.1566. Found: 667.1576.

3'-Ethyl 5',6'-dimethyl 2'-amino-5-chloro-1'-(4-methylbenzamido)-2-oxo-1'*H*-spiro[indoline-3,4'-pyridine]-3',5',6'-tricarboxylate (1h): yellow solid, 69%, m.p. 188~190℃; ^{1}H NMR (600 MHz, DMSO-d_6) δ: *cis*-isomers: 11.43 (s, 1H, NH), 10.32 (s, 1H, NH), 7.96~7.72 (m, 4H, ArH, NH_2), 7.51~7.43 (m, 1H, ArH), 7.37 (d, J = 7.2Hz, 2H, ArH), 7.17 (d, J = 7.8Hz, 1H, ArH), 6.71 (d, J = 7.8Hz, 1H, ArH), 3.78~3.73 (m, 2H, OCH_2), 3.63 (s, 3H, OCH_3), 3.40 (s, 3H, OCH_3), 2.39 (s, 3H, CH_3), 0.88 (t, J = 6.6Hz, 3H, CH_3); *trans*-isomer: 11.24 (s, 1H, NH), 10.11 (s, 1H, NH), 7.31 (d, J = 7.2Hz, 2H, ArH), 7.11 (d, J = 7.8Hz, 1H, ArH), 3.56 (s, 3H, OCH_3), 3.31 (s, 3H, OCH_3), 2.37 (s, 3H, CH_3). *cis/trans* isomers: 5:1. ^{13}C NMR (150 MHz, DMSO-d_6) δ: 180.5, 167.6, 166.9, 163.7, 162.2, 153.1, 143.3, 142.9, 141.6, 138.9, 129.7, 129.1, 128.9, 128.2, 127.9, 127.7, 127.3, 124.7, 124.0, 121.6, 120.4, 112.8, 109.5, 106.2, 77.4, 58.9, 52.9, 51.7, 50.1, 45.7, 21.1, 13.1; IR (KBr) υ: 3410, 2954, 1713, 1657, 1617, 1482, 1436, 1373, 1300, 1251, 1216, 1182, 1141, 1105, 1021, 890, 818, 752 cm^{-1}; MS (*m*/*z*): HRMS (ESI) Calcd. for $C_{27}H_{25}ClN_4NaO_8$ ([M+Na]$^+$): 591.1253. Found: 591.1246.

3'-Ethyl 5',6'-dimethyl 2'-amino-1-benzyl-5-chloro-1'-(4-methylbenzamido)-2-oxo-1'*H*-spiro-[indoline-3,4'-pyridine]-3',5',6'-tricarboxylate (1i): white solid, 70%, m.p. 188~190℃; ^{1}H NMR (600 MHz, DMSO-d_6) δ: *cis*-isomer: 11.48 (s, 1H, NH), 7.80 (brs, 2H, NH_2), 7.76 (s, 1H, ArH), 7.54 (brs, 3H, ArH), 7.37 (brs, 5H, ArH), 7.3~7.29 (m, 1H, ArH), 7.26~7.25 (m, 1H, ArH), 6.99~6.96 (m, 1H, ArH), 4.91 (d, J = 15.0Hz, 1H, CH_2), 4.71 (d, J = 15.0Hz, 1H, CH_2), 3.84~3.80 (m, 2H, OCH_2), 3.62 (s, 3H, OCH_3), 3.13 (s, 3H, OCH_3), 2.39 (s, 3H, CH_3), 0.62~0.55 (m, 3H, CH_3); *trans*-isomer: 11.27 (s, 1H, NH), 3.56 (s, 3H, OCH_3), 3.04 (s, 3H, OCH_3). *cis/trans* isomers: 6.5:1. ^{13}C NMR (150 MHz, DMSO-d_6) δ: 178.9, 167.4, 166.7, 163.7, 162.1, 153.2, 143.7, 143.0, 142.4, 138.3, 136.6, 129.1, 128.6, 128.4, 128.1, 127.9, 127.6, 127.4, 125.7, 123.8, 109.0, 105.6, 77.1, 58.6, 52.9, 51.6, 49.5, 44.2, 21.1, 13.8; IR (KBr) υ: 3371, 3318, 3271, 2946, 1749, 1720, 1694, 1662, 1607, 1478, 1432, 1370, 1331, 1293, 1293, 1249, 1203, 1177, 1140, 1113, 1091, 1023, 941, 885, 815, 781, 745 cm^{-1}; MS (*m*/*z*): HRMS (ESI) Calcd. for $C_{34}H_{31}ClN_4NaO_8$ ([M+Na]$^+$): 681.1723. Found: 681.1716.

3'-Ethyl 5',6'-dimethyl 2'-amino-5-methyl-2-oxo-1'-(picolinamido)-1'*H*-spiro[indoline-3,4'-pyridine]-3',5',6'-tricarboxylate (1j): White solid, 80%, m.p. 178~180℃; ^{1}H NMR (600 MHz, DMSO-d_6) δ: *cis*-isomer: 11.49 (s, 1H, NH), 10.01 (s, 1H, NH), 8.74~8.71 (m, 1H, ArH), 8.11~8.09 (m, 1H, ArH), 8.07~8.06 (m, 1H, ArH), 7.73~7.70 (m, 1H, ArH), 7.55 (brs, 2H, NH_2), 7.32 (s, 1H, ArH), 6.93~6.88 (m, 1H, ArH), 6.59~6.55 (m, 1H, ArH), 3.75~3.69 (m, 2H, OCH_2),

3.58 (s, 3H, OCH_3), 3.37 (s, 3H, OCH_3), 2.26 (s, 3H, CH_3), 0.86 (t, J = 7.2Hz, 3H, CH_3); *trans*-isomer: 11.37 (s, 1H, NH), 9.98 (s, 1H, NH), 7.83 (brs, 2H, NH_2), 3.49 (s, 3H, OCH_3), 3.27 (s, 3H, OCH_3), 2.23 (s, 3H, CH_3). *cis/trans* isomers: 5:1. ^{13}C NMR (150 MHz, DMSO-d_6) δ: 180.7, 167.8, 164.6, 163.9, 162.3, 152.9, 148.8, 148.6, 148.1, 142.6, 140.2, 137.9, 137.1, 129.3, 127.9, 127.7, 124.7, 122.9, 107.8, 107.0, 78.4, 58.7, 52.7, 52.5, 51.5, 51.4, 50.0, 20.9, 13.1; IR (KBr) υ: 3327, 2983, 2952, 1746, 1710, 1662, 1617, 1494, 1470, 1432, 1371, 1295, 1209, 1148, 1106, 1038, 887, 853, 813, 753 cm^{-1}; MS (*m/z*): HRMS (ESI) Calcd. for $C_{26}H_{25}N_5NaO_8$ ($[M+Na]^+$): 558.1595. Found: 558.1596.

3'-Ethyl 5',6'-dimethyl 2'-amino-5-chloro-2-oxo-1'-(picolinamido)-1'*H*-spiro[indoline-3,4'-pyridine]-3',5',6'-tricarboxylate (1k): White solid, 82%, m.p. 202~204℃; ^{1}H NMR (600 MHz, DMSO-d_6) δ: *cis*-isomer: 6.5:1. 11.58 (s, 1H, NH), 10.30 (s, 1H, NH), 8.74 (s, 1H, ArH), 8.09~8.08 (m, 2H, ArH), 7.72 (brs, 2H, NH_2), 7.57~7.48 (m, 2H, ArH), 7.20~7.14 (m, 1H, ArH), 6.71 (d, J = 7.8Hz, 1H, ArH), 3.79~3.74 (m, 2H, OCH_2), 3.60 (s, 3H, OCH_3), 3.40 (s, 3H, OCH_3), 0.89 (t, J = 7.2Hz, 3H, CH_3); *trans*-isomer: 11.28 (s, 1H, NH), 10.26 (s, 1H, NH), 8.72 (s, 1H, ArH), 7.91 (brs, 2H, NH_2), 3.49 (s, 3H, OCH_3), 3.37 (s, 3H, OCH_3). *cis/trans* isomers: 4:1. ^{13}C NMR (150 MHz, DMSO-d_6) δ: 180.7, 167.8, 164.6, 163.9, 162.3, 152.9, 148.8, 148.6, 148.1, 142.6, 140.2, 137.9, 137.1, 129.3, 127.9, 127.7, 124.7, 122.9, 107.8, 107.0, 78.4, 58.7, 52.7, 52.5, 51.5, 51.4, 50.0, 20.9, 13.1; IR (KBr) υ: 3429, 3270, 3189, 2989, 2955, 2905, 1717, 1666, 1620, 1472, 1432, 1366, 1295, 1210, 1178, 1136, 1106, 1039, 992, 942, 889, 851, 819, 779, 752 cm^{-1}; MS (*m/z*): HRMS (ESI) Calcd. for $C_{25}H_{22}ClN_5NaO_8$ ($[M+Na]^+$): 578.1049. Found: 578.1054.

3'-Ethyl 5',6'-dimethyl 2'-amino-1-benzyl-5-chloro-2-oxo-1'-(picolinamido)-1'*H*-spiro-[indoline-3,4'-pyridine]-3',5',6'-tricarboxylate (1l): White solid, 68%, m.p. 228~230℃; ^{1}H NMR (600 MHz, DMSO-d_6) δ: *cis*-isomer: 11.60 (s, 1H, NH), 8.74~8.72 (m, 1H, ArH), 8.09~8.07 (m, 2H, ArH), 7.73~7.65 (m, 2H, NH_2), 7.56~7.53 (m, 3H, ArH), 7.36 (t, J = 7.2Hz, 3H, ArH), 7.29 (t, J = 7.2Hz, 1H, ArH), 7.25~7.24 (m, 1H, ArH), 6.96 (d, J = 8.4Hz, 1H, ArH), 4.91 (d, J = 15.6Hz, 1H, CH_2), 4.71 (d, J = 15.6Hz, 1H, CH_2), 3.82~3.77 (m, 1H, OCH_2), 3.59 (s, 3H, OCH_3), 3.49~3.44 (m, 1H, OCH_2), 3.15 (s, 3H, OCH_3), 0.64 (t, J = 7.2Hz, 3H, CH_3); *trans*- isomer: 11.27 (s, 1H, NH), 7.02 (d, J = 8.4Hz, 1H, ArH), 3.50 (s, 3H, OCH_3), 3.09 (s, 3H, OCH_3), 0.58 (t, J = 7.2Hz, 3H, CH_3). *cis/trans* isomers: 5:1. ^{13}C NMR (150 MHz, DMSO-d_6) δ: 178.8, 167.2, 164.8, 163.7, 162.1, 153.2, 148.9, 148.0, 143.4, 142.3, 138.2, 138.0, 136.6, 128.7, 128.6, 128.5, 128.4, 127.8, 127.7, 127.4, 127.2, 125.8, 123.8, 123.0, 109.1, 105.9, 77.8, 58.6, 52.9, 51.6, 49.5, 44.2, 13.8; IR (KBr) υ: 3294, 2956, 1708, 1655, 1572, 1490, 1437, 1327, 1230, 1176, 1096, 1028, 935, 883, 811 cm^{-1}; MS (*m/z*): HRMS (ESI) Calcd. for $C_{32}H_{28}ClN_5NaO_8$ ($[M+Na]^+$): 668.1519. Found: 668.1516.

3'-Ethyl 5',6'-dimethyl 2'-amino-1-benzyl-5-methyl-2-oxo-1'-(picolinamido)-1'*H*-spiro-[indoline-3,4'-pyridine]-3',5',6'-tricarboxylate (1m): White solid, 81%, m.p. 240~242℃; ^{1}H NMR (600 MHz, DMSO-d_6) δ: *cis*-isomer: 11.55 (s, 1H, NH), 8.74~8.72 (m, 1H, ArH), 8.10~8.06 (m, 2H, ArH), 7.90~7.64 (m, 3H, ArH, NH_2), 7.54 (d, J = 7.2Hz, 2H, ArH), 7.38~7.34 (m, 3H, ArH), 7.28 (t, J = 7.2Hz, 1H, ArH), 6.97 (d, J = 7.2Hz, 1H, ArH), 6.79 (d, J = 7.8Hz, 1H, ArH), 4.88 (d, J = 15.6Hz, 1H, CH_2), 4.66 (d, J = 15.6Hz, 1H, CH_2), 3.77~3.71 (m, 1H, OCH_2), 3.57 (s, 3H, OCH_3), 3.48~3.44 (m, 1H, OCH_2), 3.09 (s, 3H, OCH_3), 2.27 (s, 3H, CH_3), 0.61 (t, J = 7.2Hz, 3H, CH_3); *trans*-isomer: 11.39 (s, 1H, NH), 6.75 (d, J = 7.8Hz, 1H, ArH), 3.50 (s, 3H, OCH_3), 3.02

(s, 3H, OCH_3), 2.24 (s, 3H, CH_3), 0.55 (t, J = 7.2Hz, 3H, CH_3). *cis/trans* isomers: 3:1. ^{13}C NMR (150 MHz, DMSO-d_6) δ: 179.1, 167.6, 164.6, 163.9, 162.2, 153.1, 148.8, 148.1, 143.0, 141.2, 137.9, 137.1, 136.4, 130.5, 128.7, 128.6, 128.4, 128.3, 128.0, 127.7, 127.3, 124.5, 123.0, 107.3, 106.3, 78.1, 58.4, 52.7, 51.4, 49.4, 44.2, 20.8, 13.7; IR (KBr) υ: 3293, 3040, 1749, 1699, 1663, 1611, 1434, 1427, 1365, 1334, 1296, 1263, 1175, 1142, 1105, 1028, 942, 890, 817, 781 cm^{-1}; MS (*m*/*z*): HRMS (ESI) Calcd. for $C_{33}H_{31}N_5NaO_8$ ([M+Na]$^+$): 648.2065. Found: 648.2064.

Dimethyl 2'-amino-1'-benzamido-1-benzyl-3'-cyano-2-oxo-1'*H*-spiro[indoline-3,4'-pyridine]-5',6'-dicarboxylate (1a):

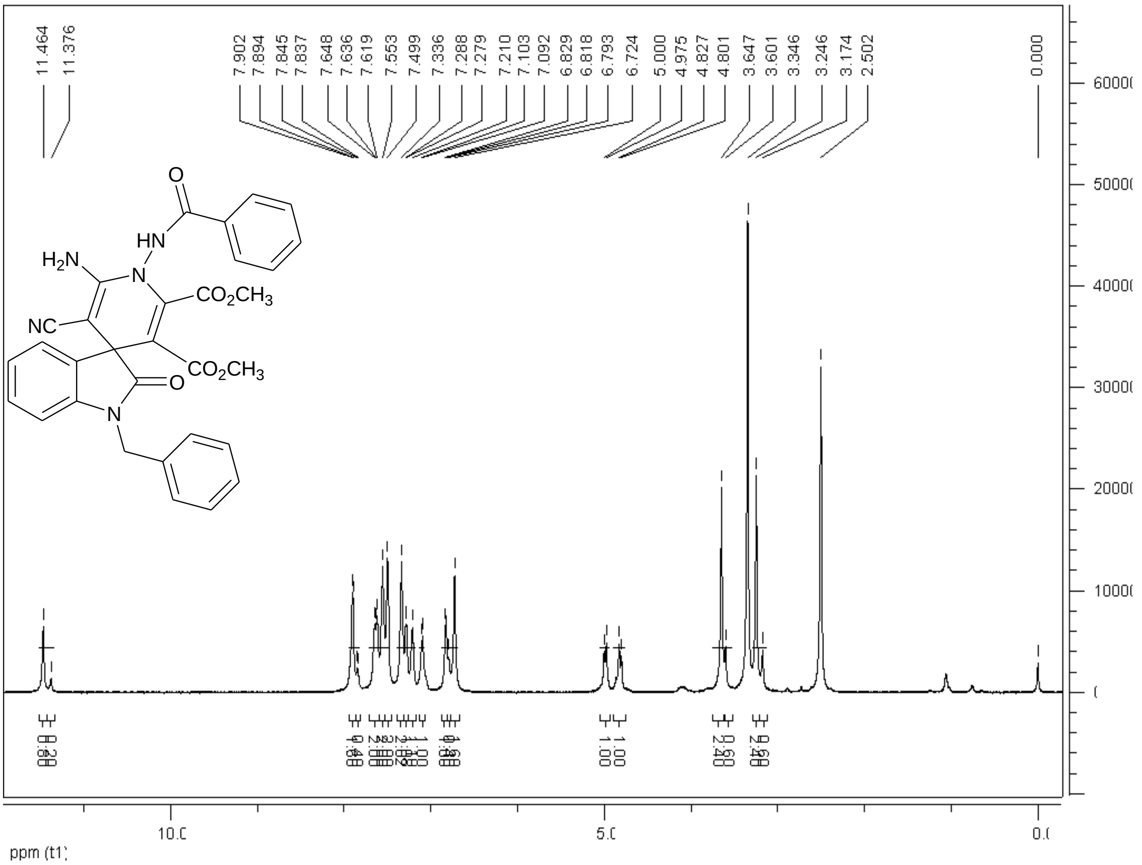

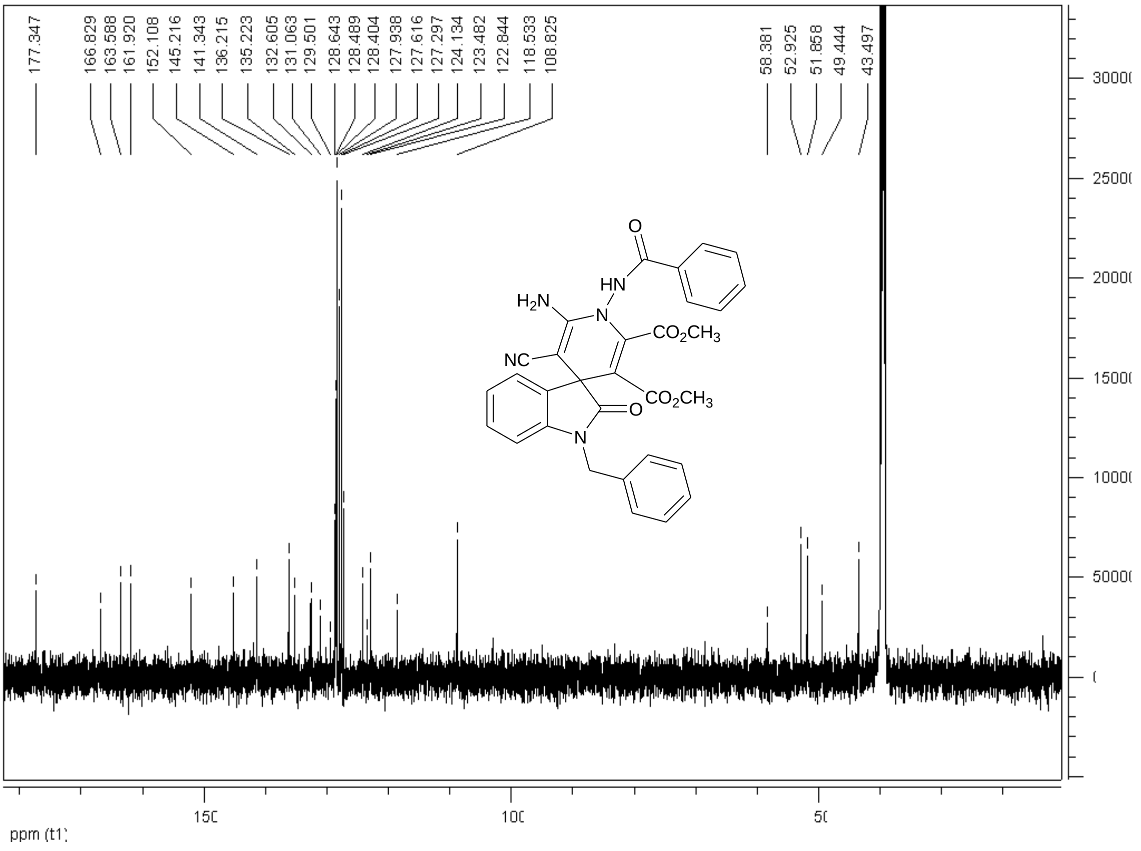

Dimethyl 2'-amino-1'-benzamido-1-benzyl-3'-cyano-5-fluoro-2-oxo-1'*H*-spiro[indoline-3,4'-pyridine]-5',6'-dicarboxylate (1b):

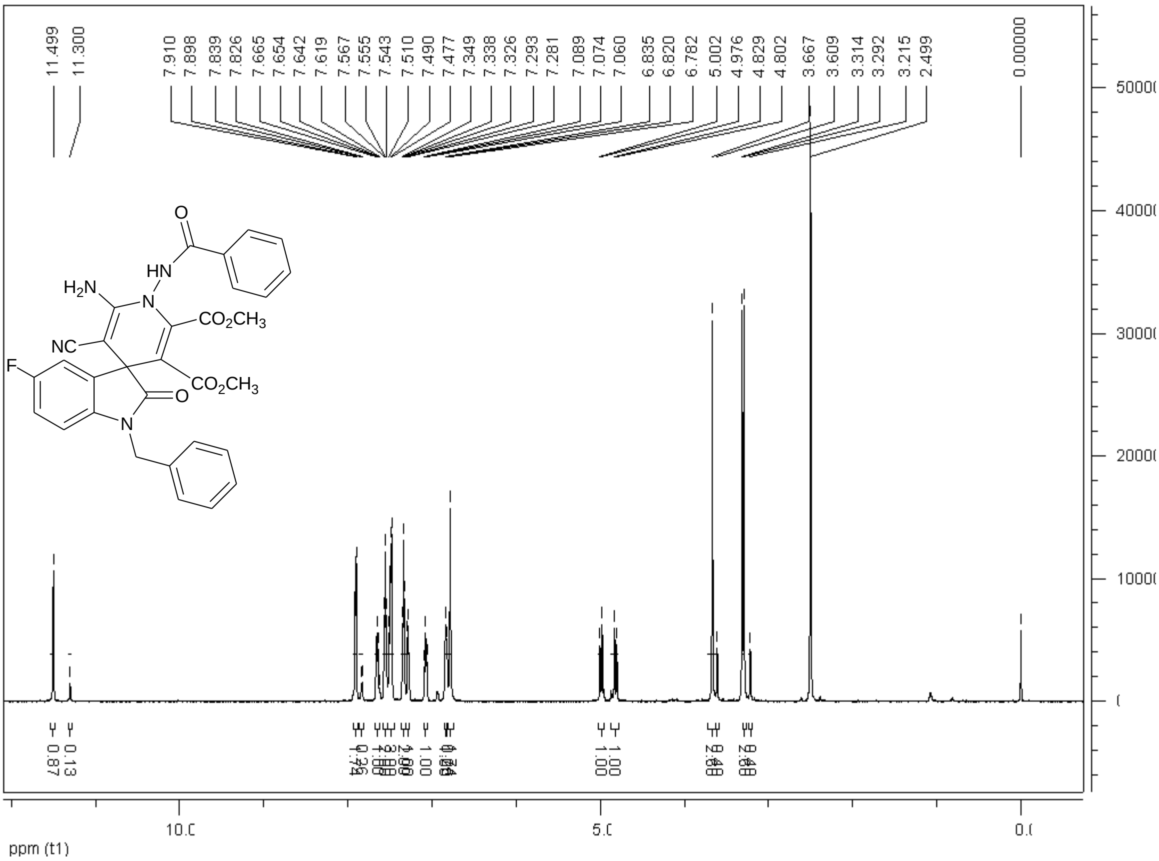

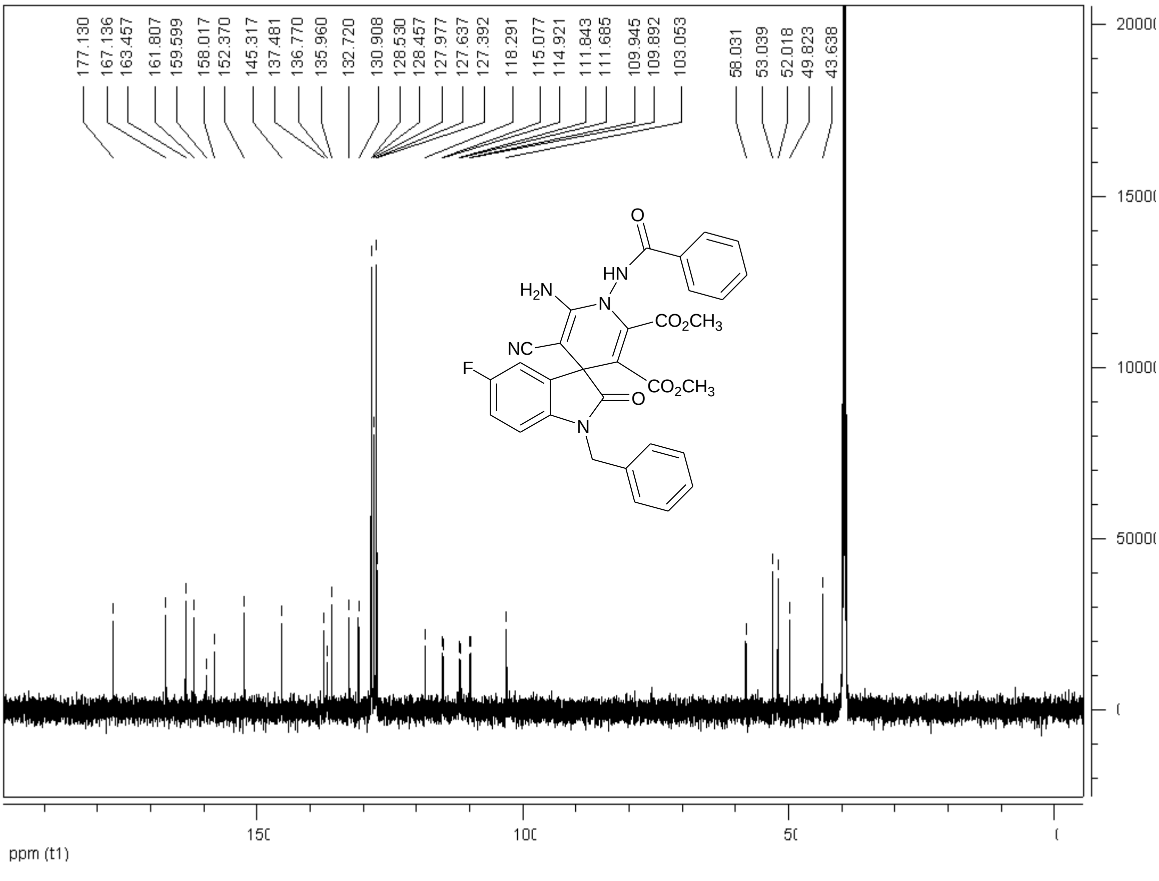

Dimethyl 2'-amino-3'-cyano-1'-(4-methylbenzamido)-2-oxo-1'*H*-spiro[indoline-3,4'-pyridine]-5',6'-dicarboxylate (1c):

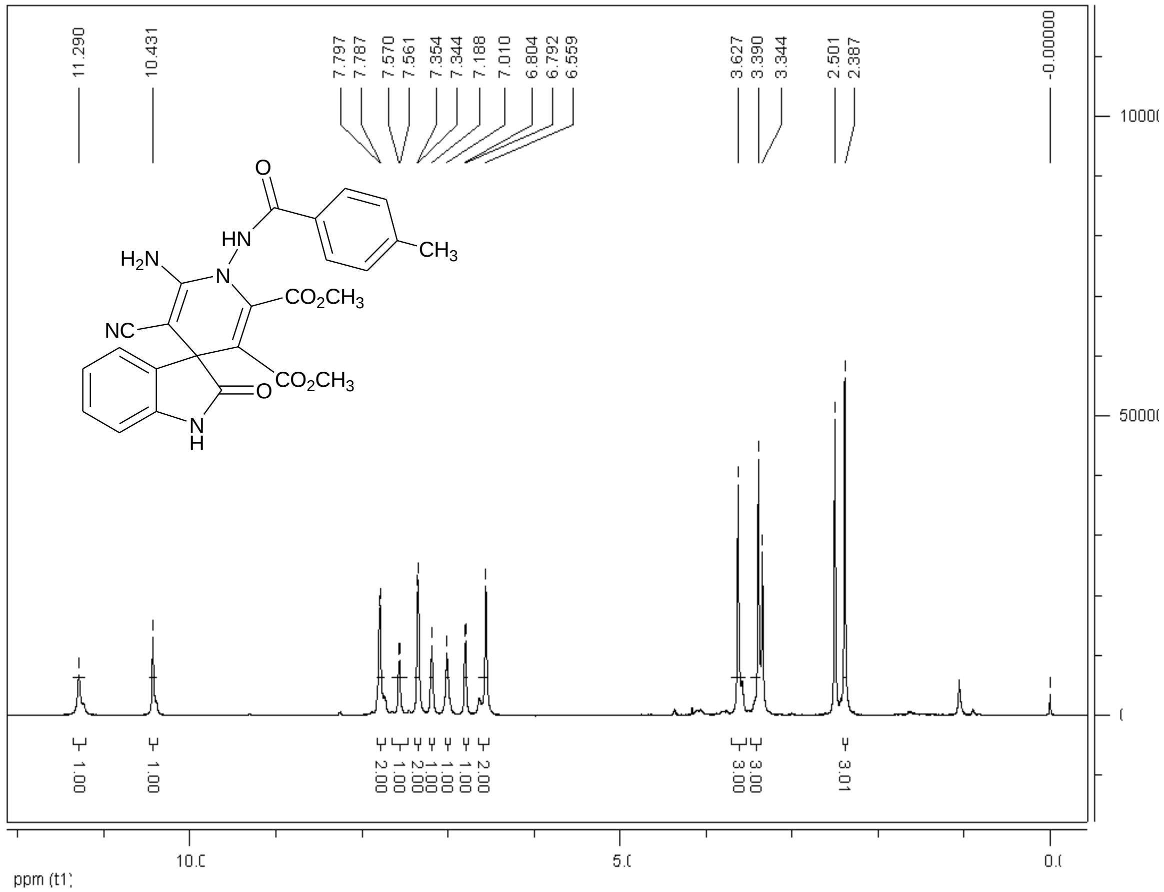

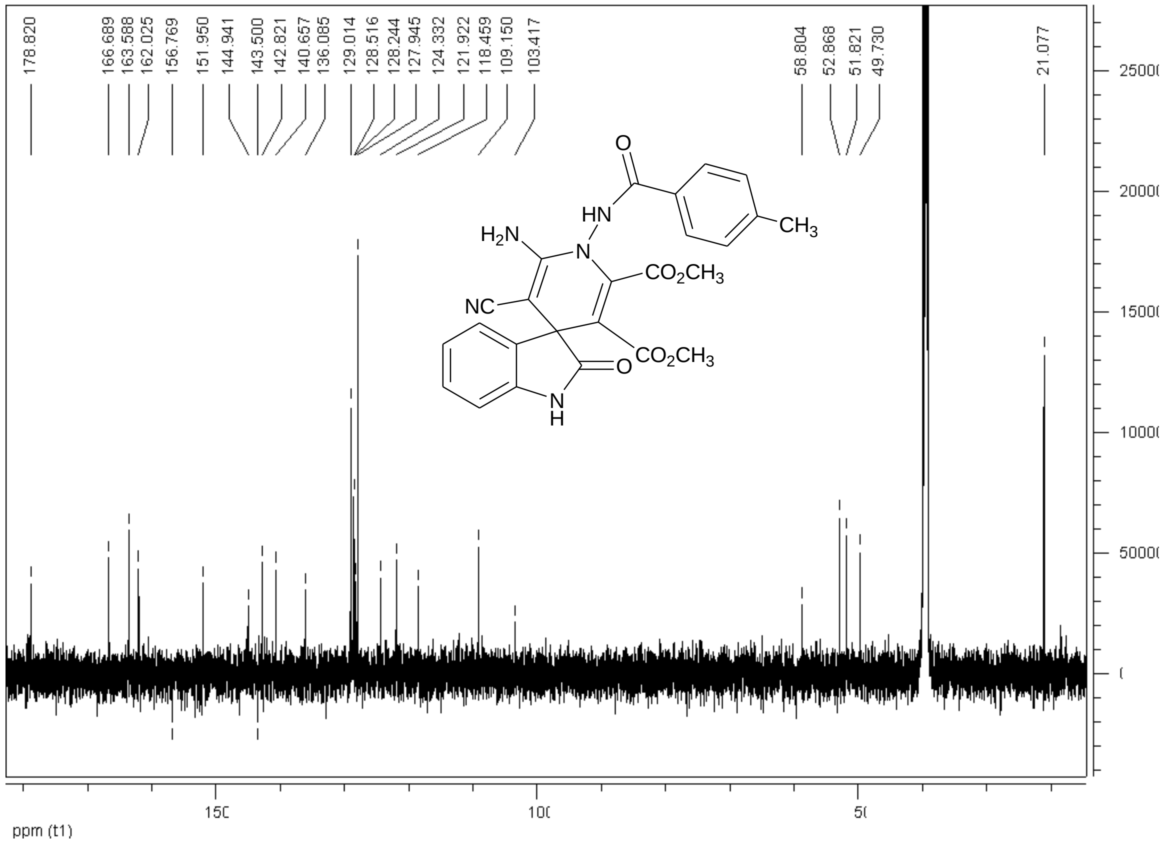

Dimethyl 2'-amino-1-benzyl-3'-cyano-5-fluoro-1'-(4-methylbenzamido)-2-oxo-1'*H*-spiro[indoline-3,4'-pyridine]-5',6'-dicarboxylate (1d):

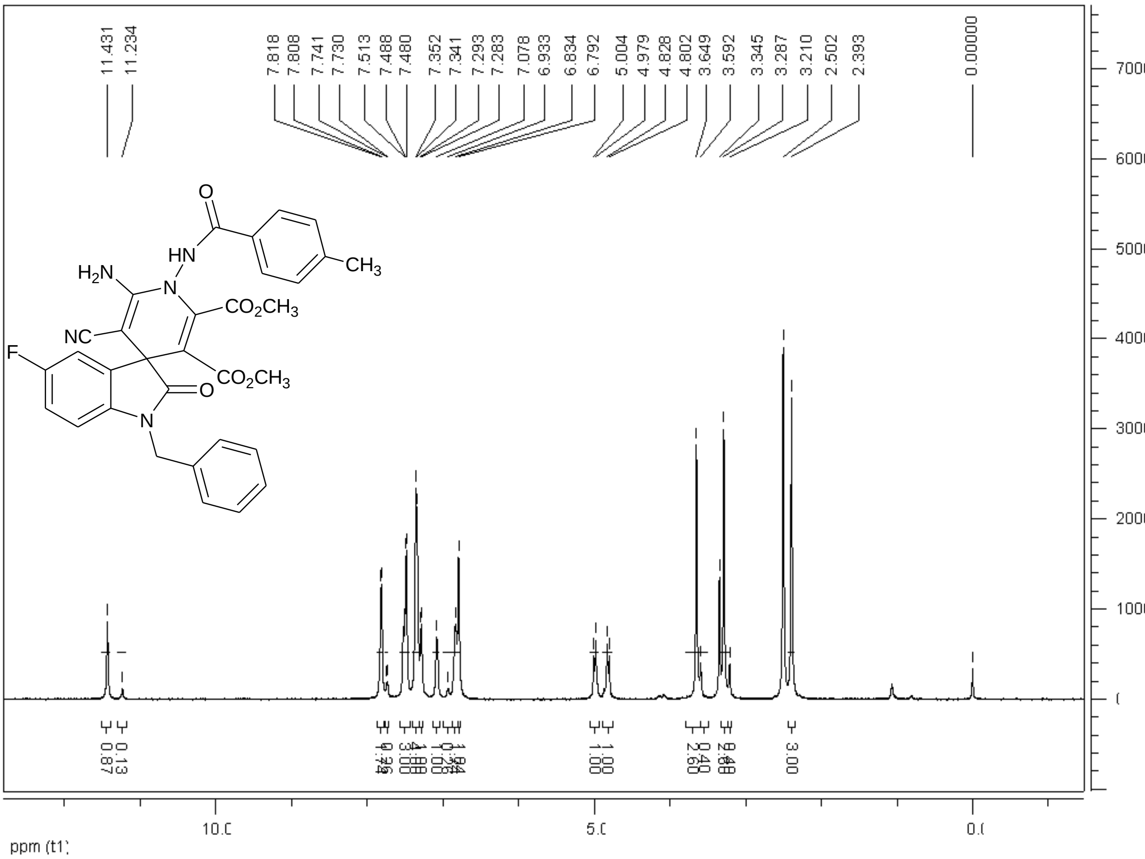

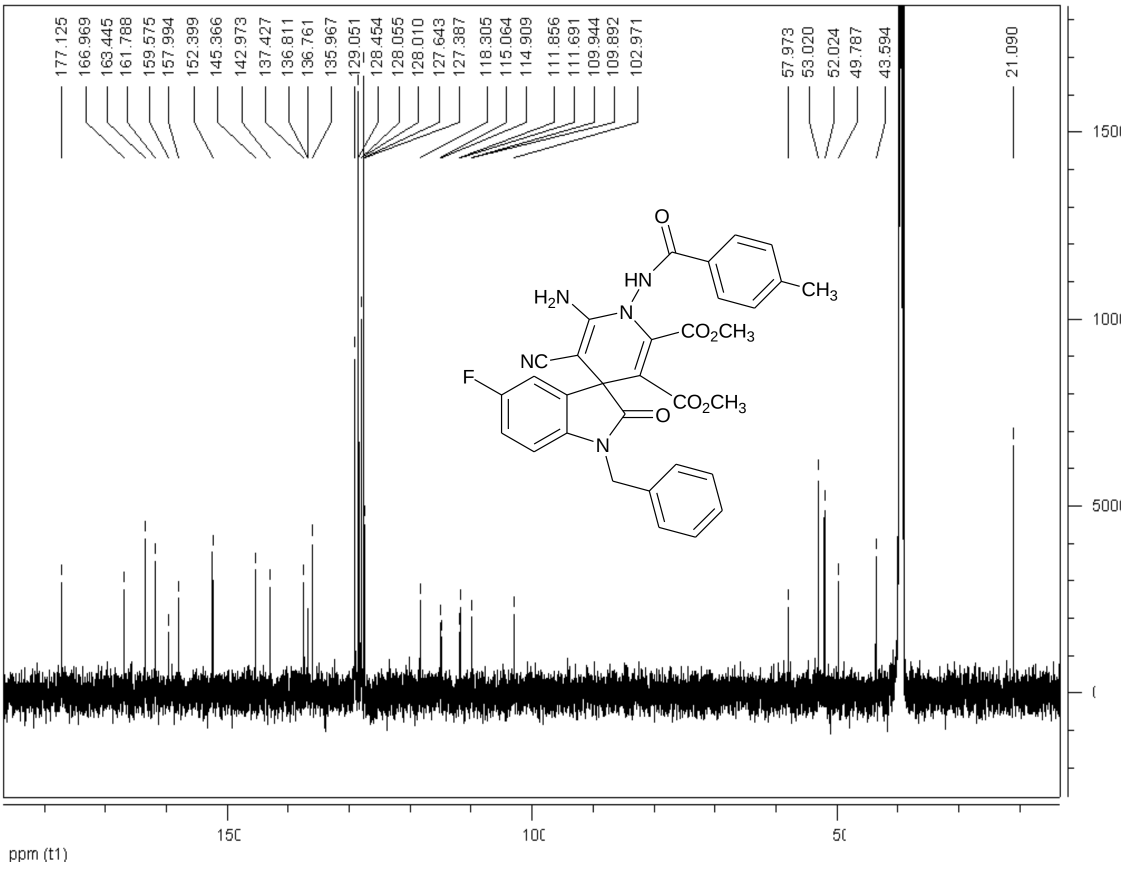

3'-Ethyl 5',6'-dimethyl 2'-amino-1'-benzamido-2-oxo-1'*H*-spiro[indoline-3,4'-pyridine]-3',5',6'-tricarboxylate (1e):

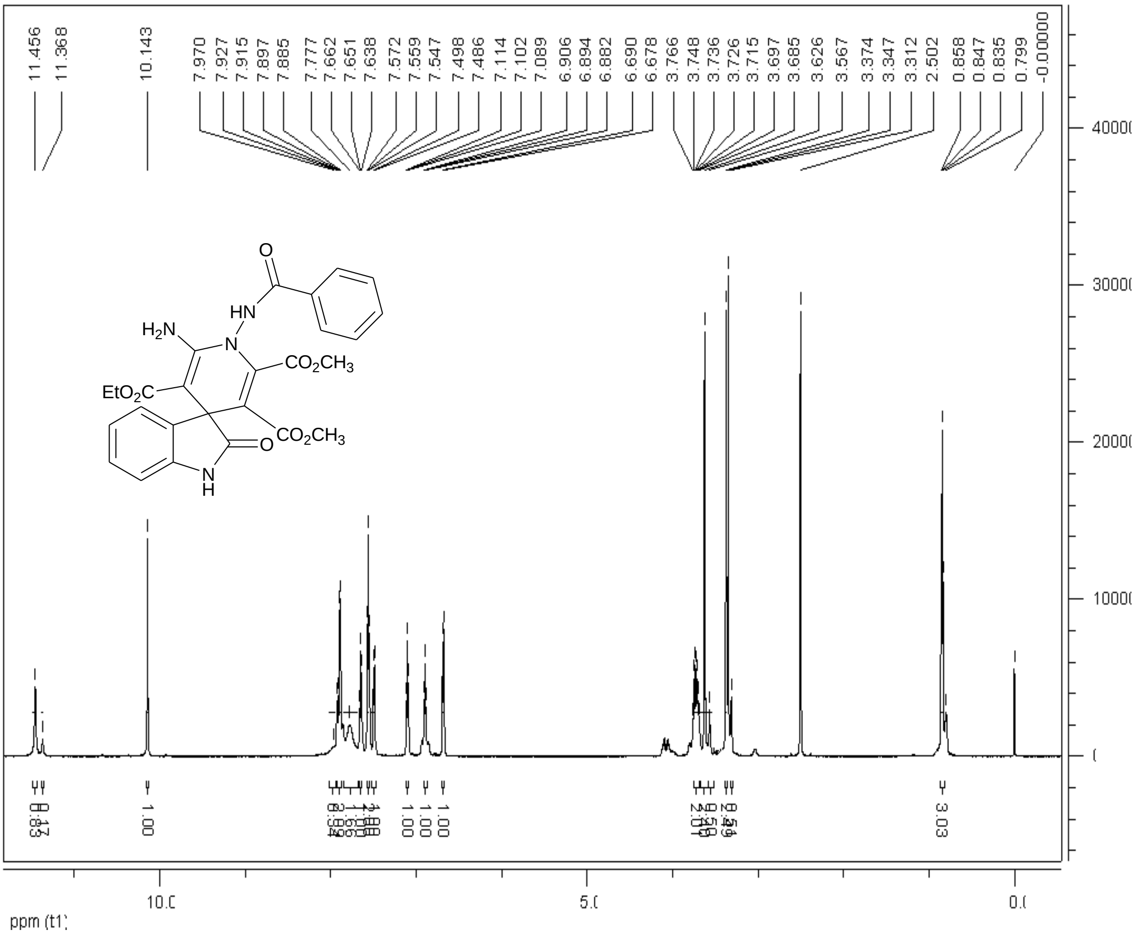

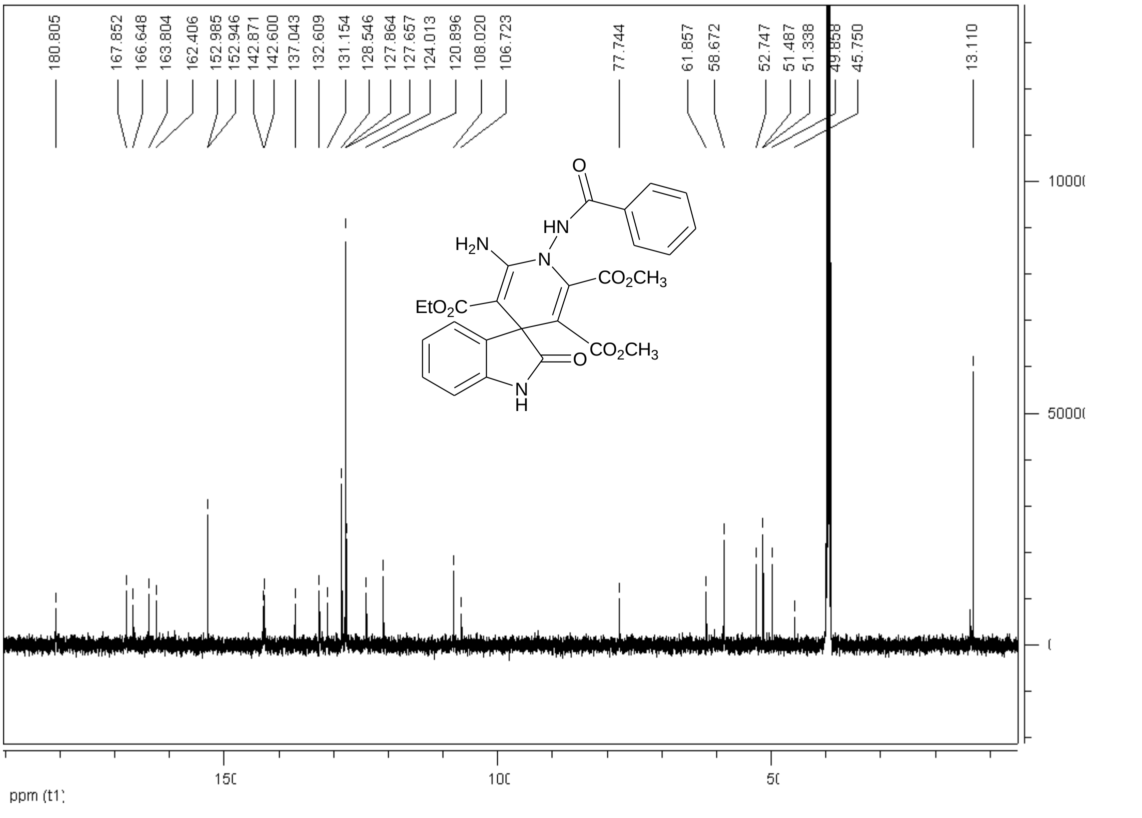

3'-Ethyl 5',6'-dimethyl 2'-amino-1'-benzamido-5-chloro-2-oxo-1'*H*-spiro[indoline-3,4'-pyridine]-3',5',6'-tricarboxylate (1f):

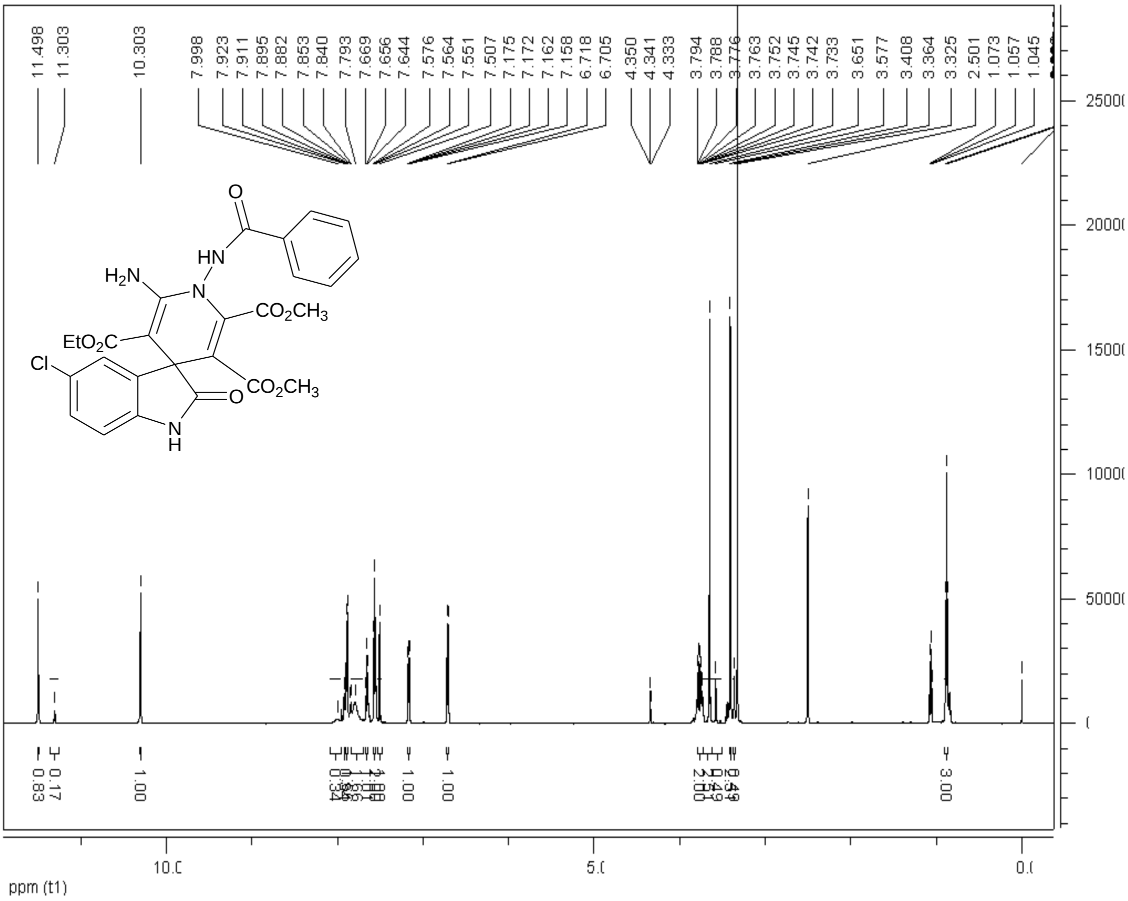

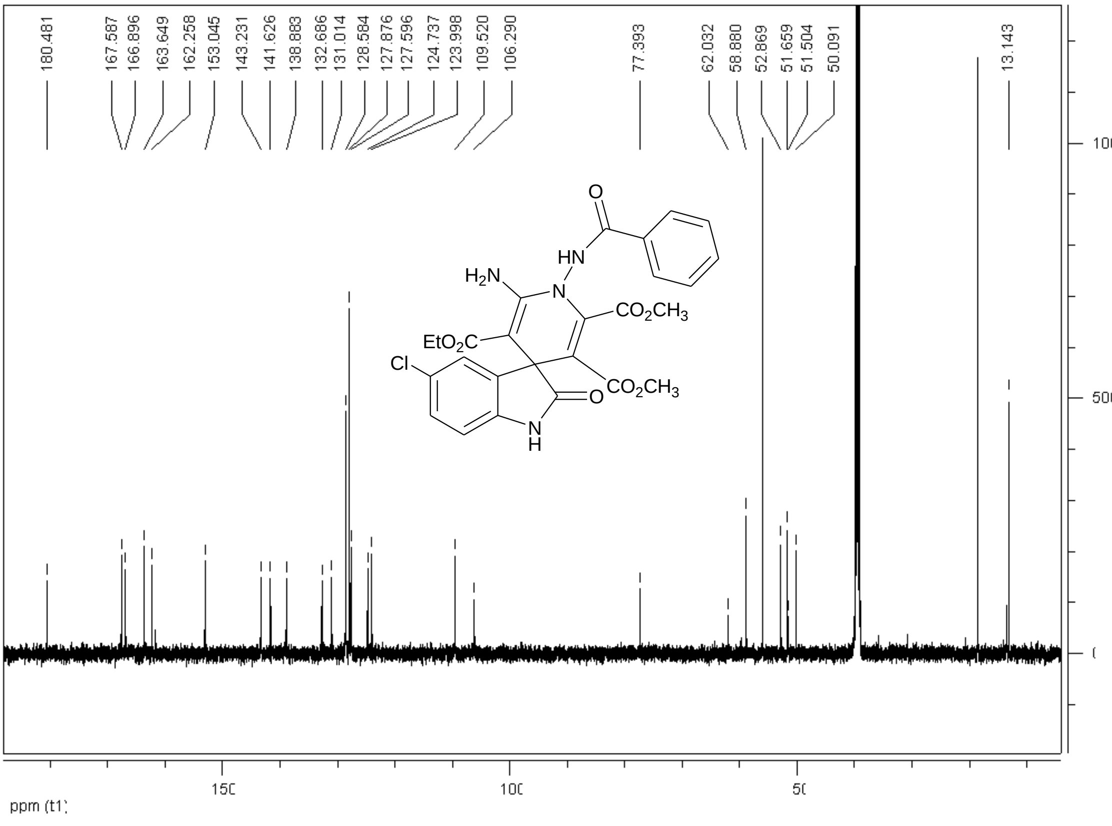

3'-Ethyl 5',6'-dimethyl 2'-amino-1'-benzamido-1-benzyl-5-chloro-2-oxo-1'*H*-spiro[indoline-3,4'-pyridine]-3',5',6'-tricarboxylate (1g):

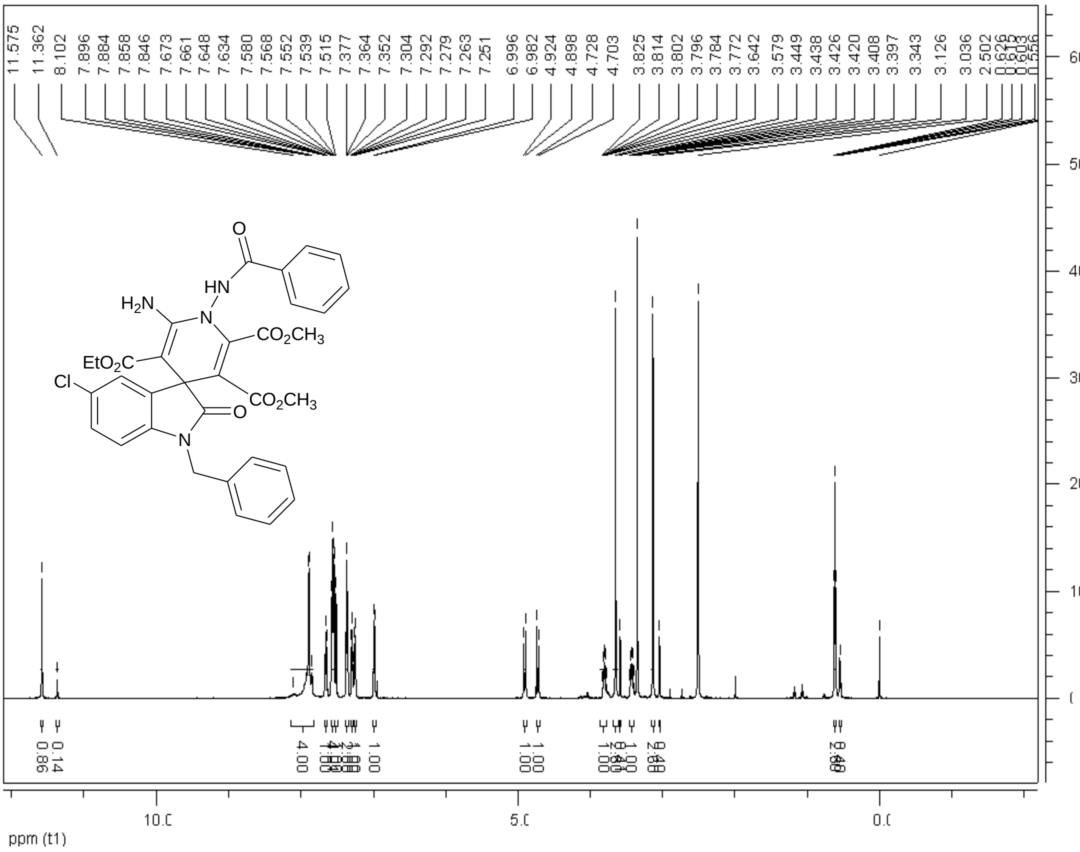

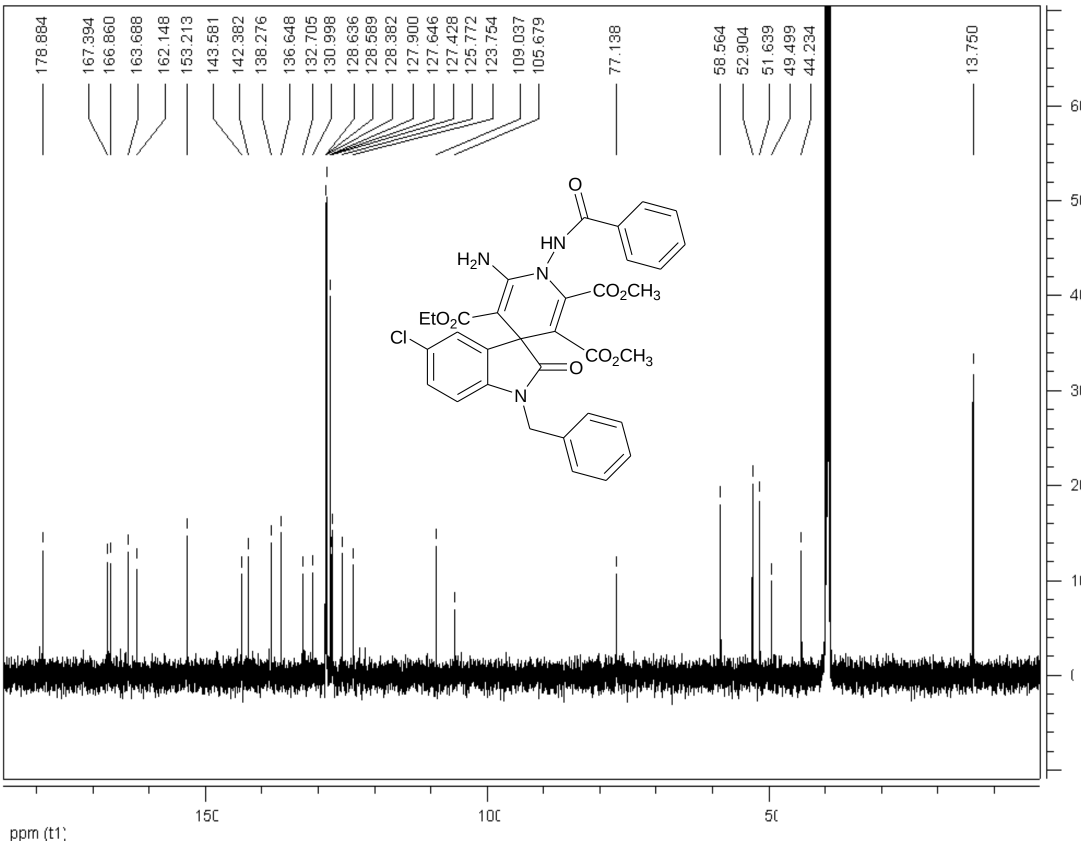

3'-Ethyl 5',6'-dimethyl 2'-amino-5-chloro-1'-(4-methylbenzamido)-2-oxo-1'*H*-spiro-[indoline-3,4'-pyridine]-3',5',6'-tricarboxylate (1h):

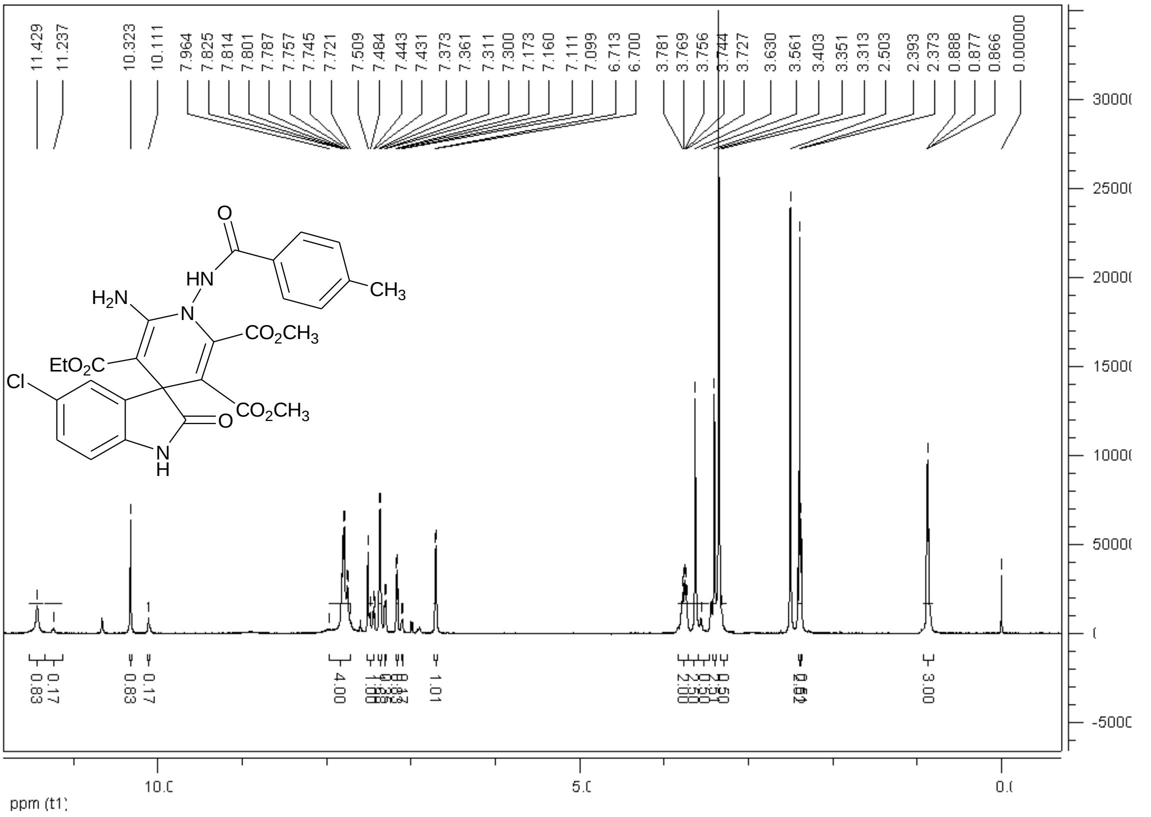

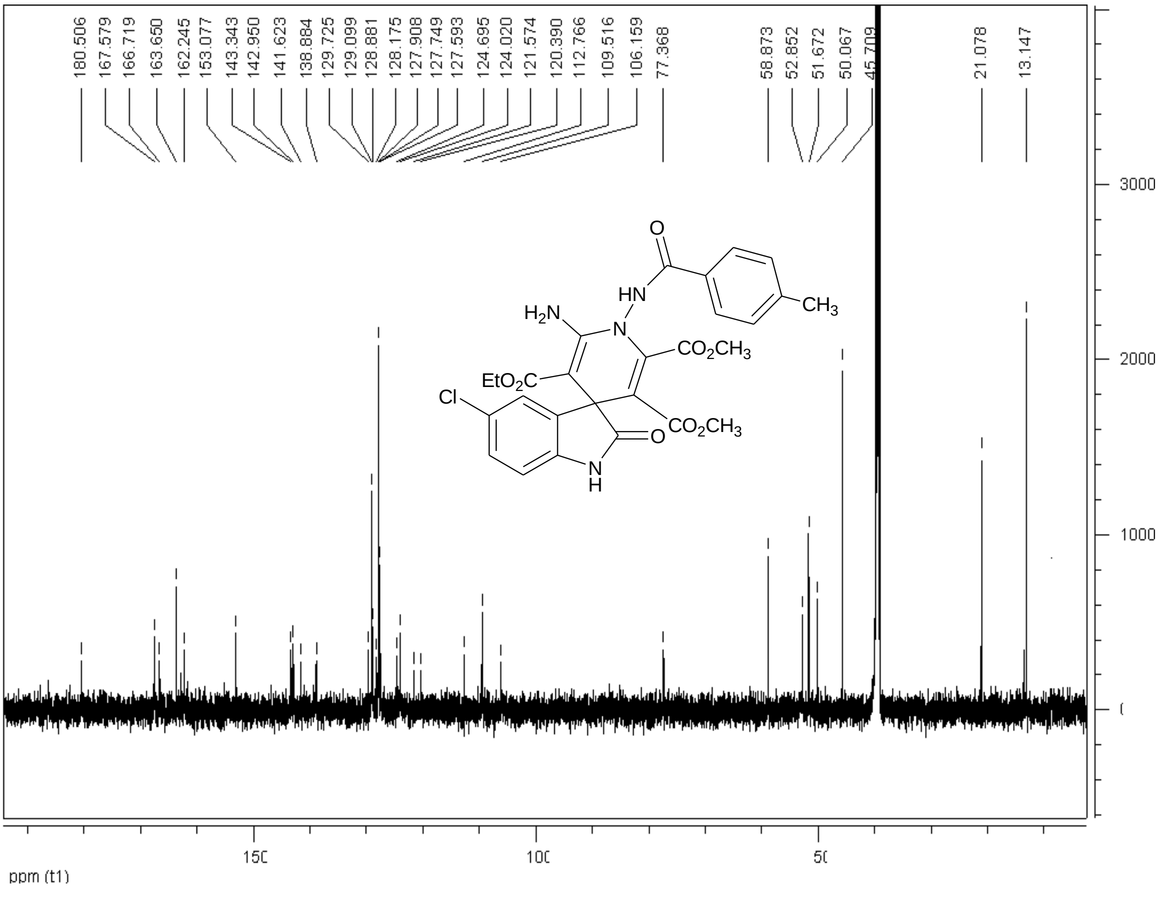

3'-Ethyl 5',6'-dimethyl 2'-amino-1-benzyl-5-chloro-1'-(4-methylbenzamido)-2-oxo-1'*H*-spiro [indoline-3,4'-pyridine]-3',5',6'-tricarboxylate (1i):

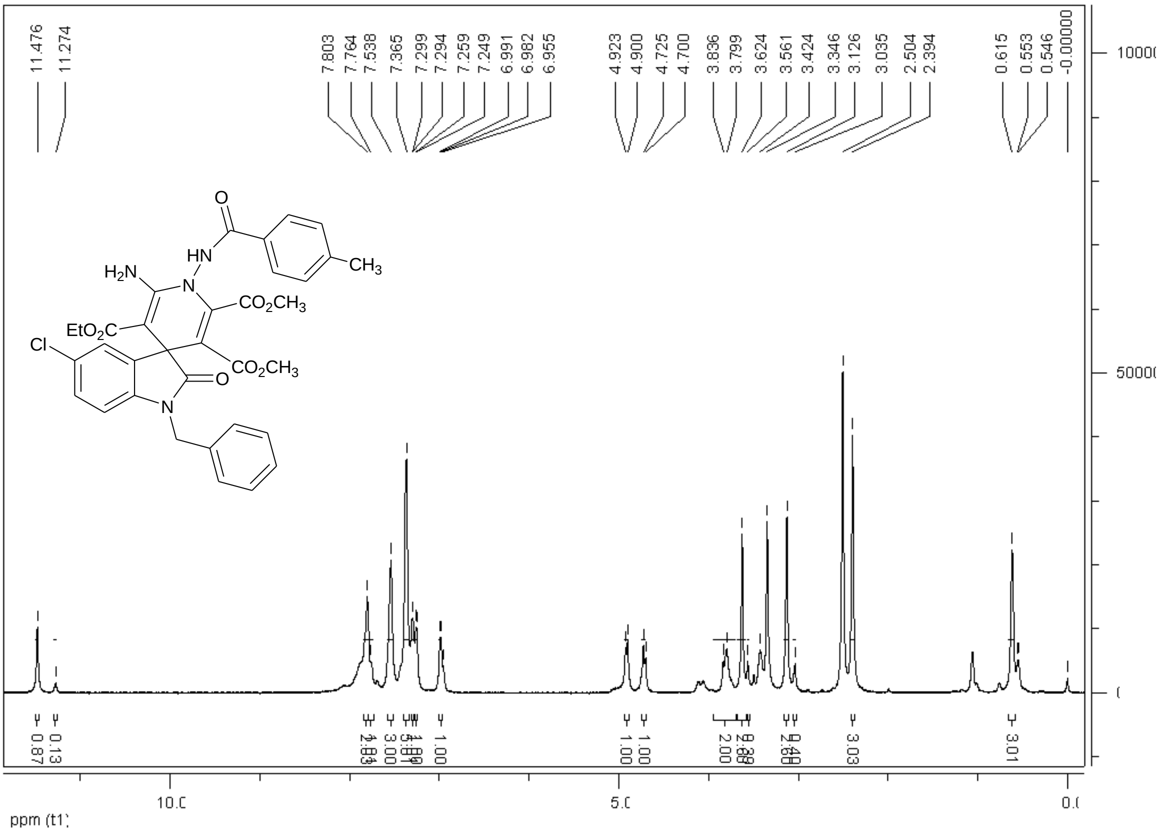

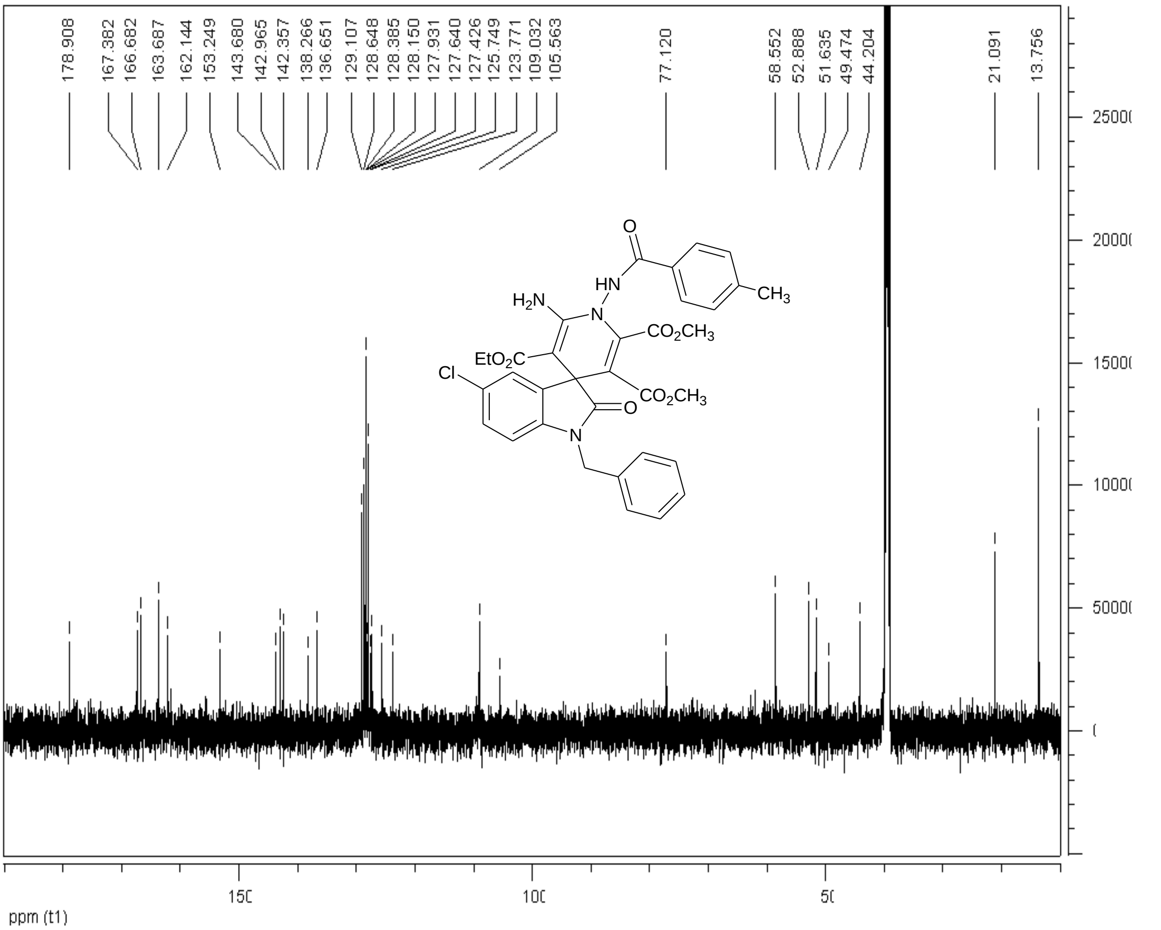

3'-Ethyl 5',6'-dimethyl 2'-amino-5-methyl-2-oxo-1'-(picolinamido)-1'*H*-spiro[indoline-3,4'-pyridine]-3',5',6'-tricarboxylate (1j):

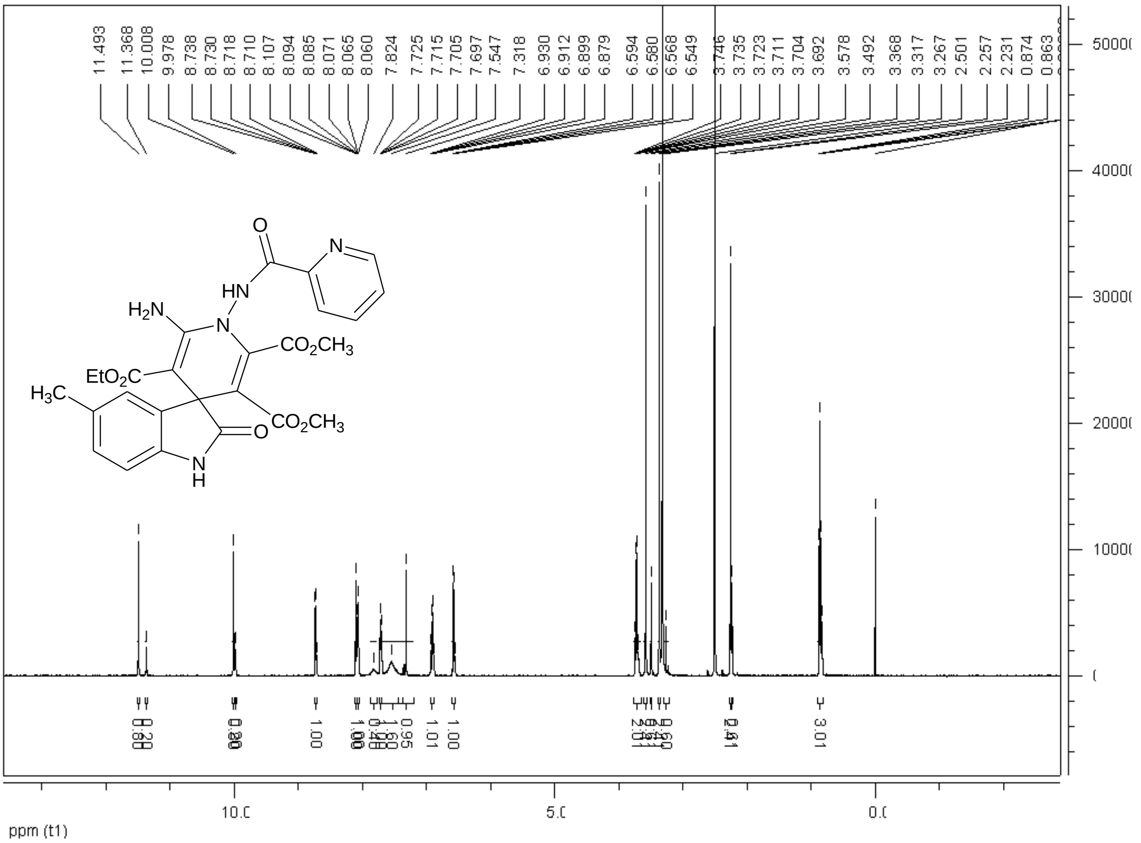

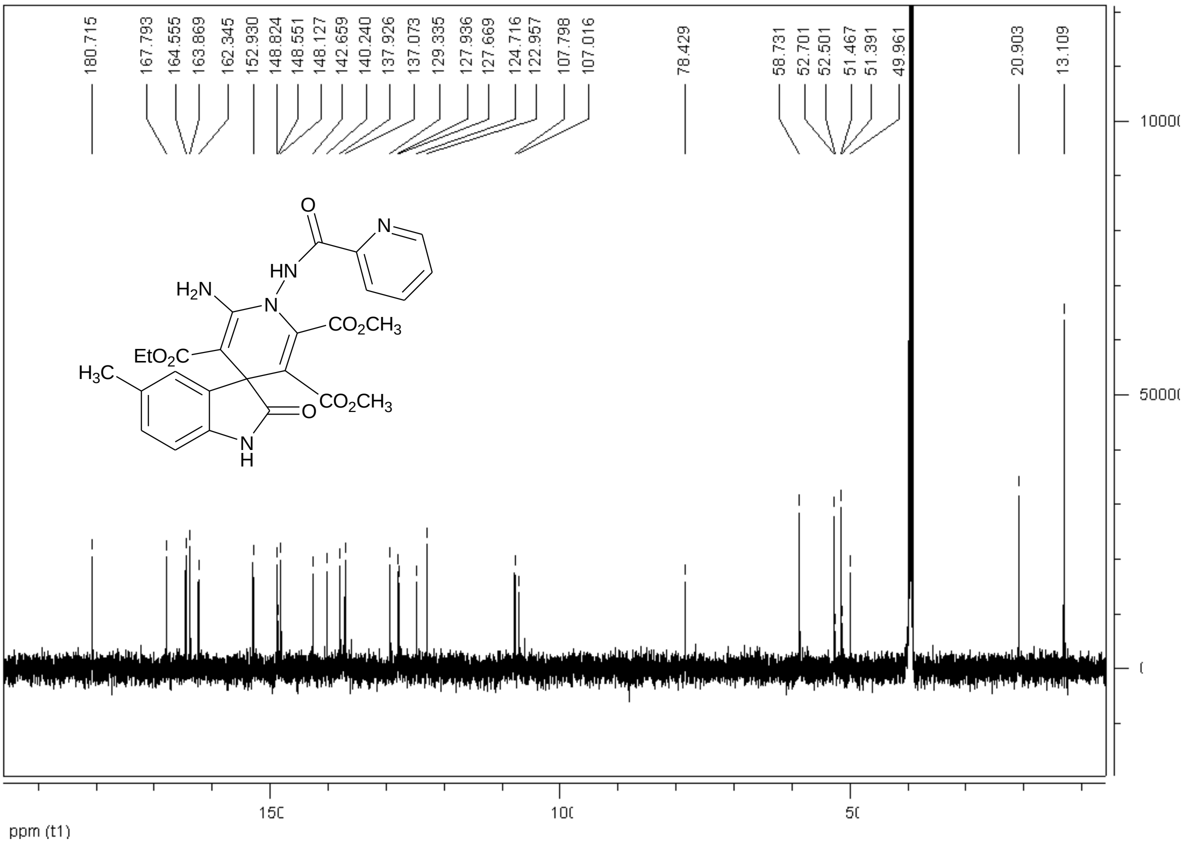

3'-Ethyl 5',6'-dimethyl 2'-amino-5-chloro-2-oxo-1'-(picolinamido)-1'*H*-spiro[indoline-3,4'-pyridine]-3',5',6'-tricarboxylate (1k):

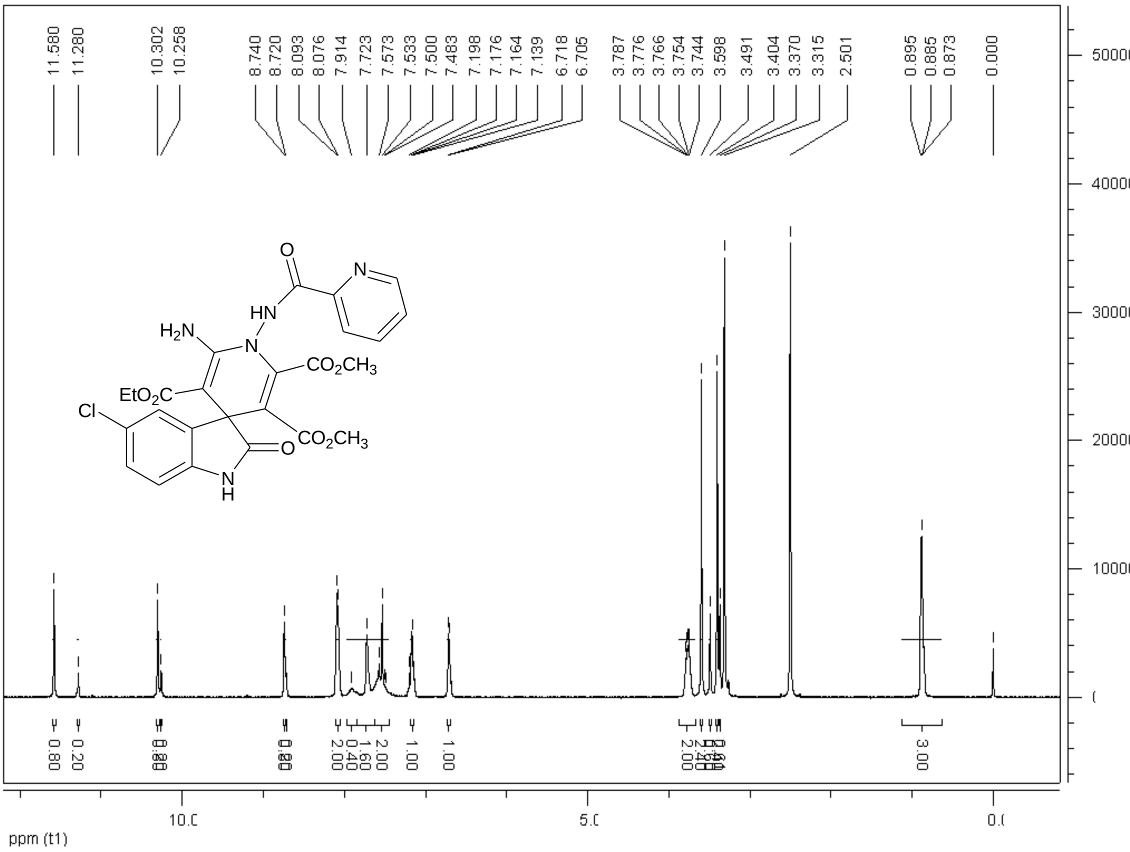

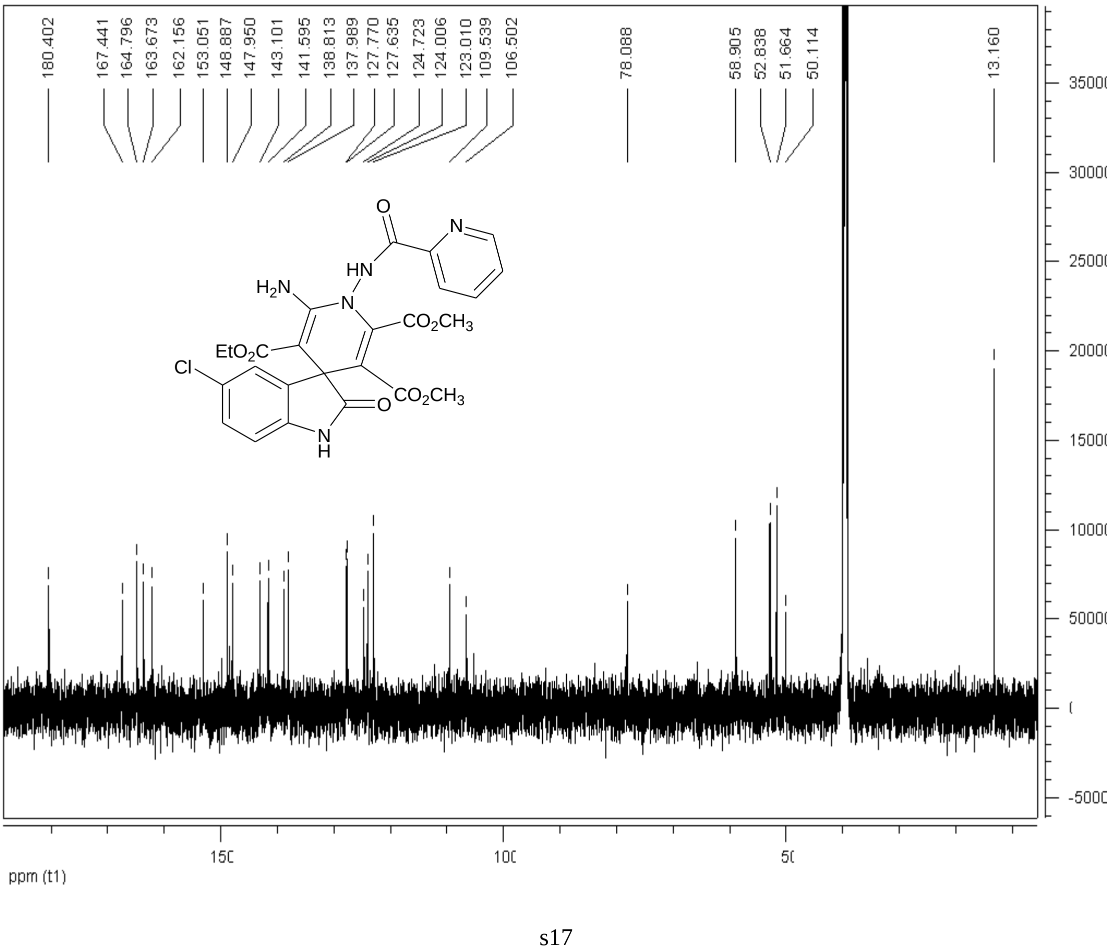

3'-Ethyl 5',6'-dimethyl 2'-amino-1-benzyl-5-chloro-2-oxo-1'-(picolinamido)-1'*H*-spiro[indoline-3,4'-pyridine]-3',5',6'-tricarboxylate (1l):

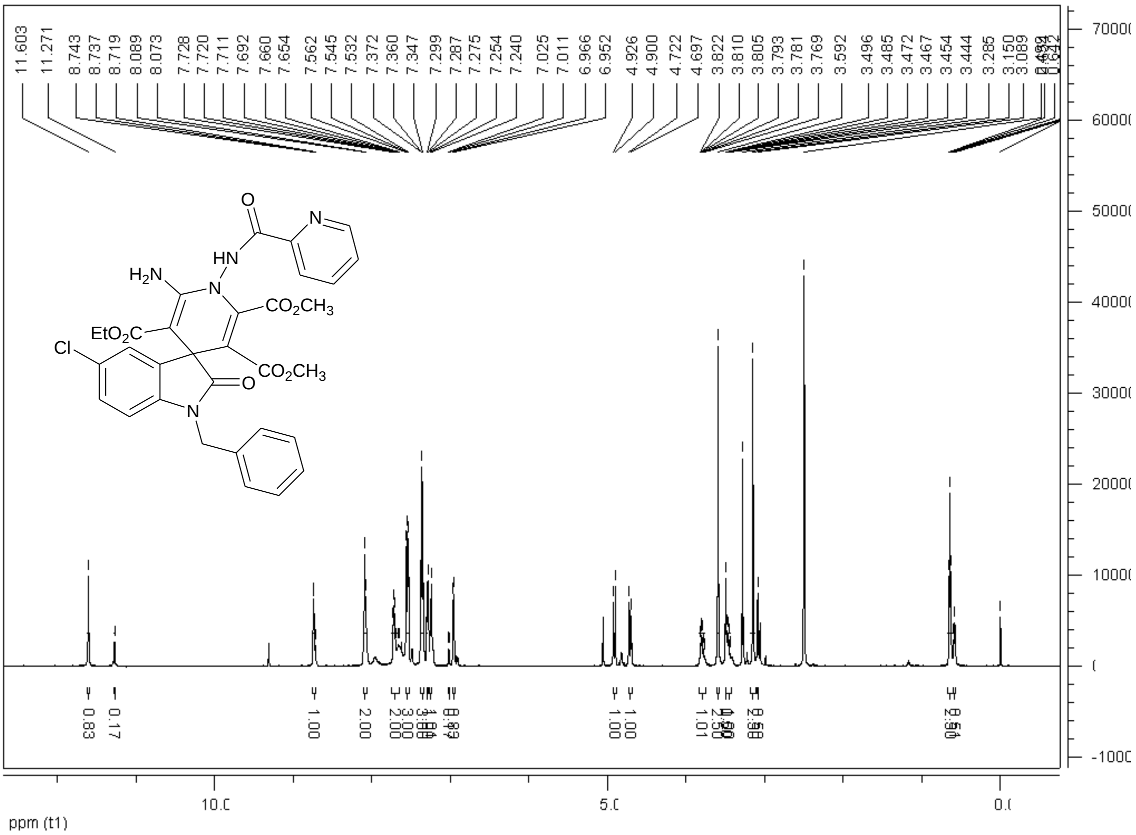

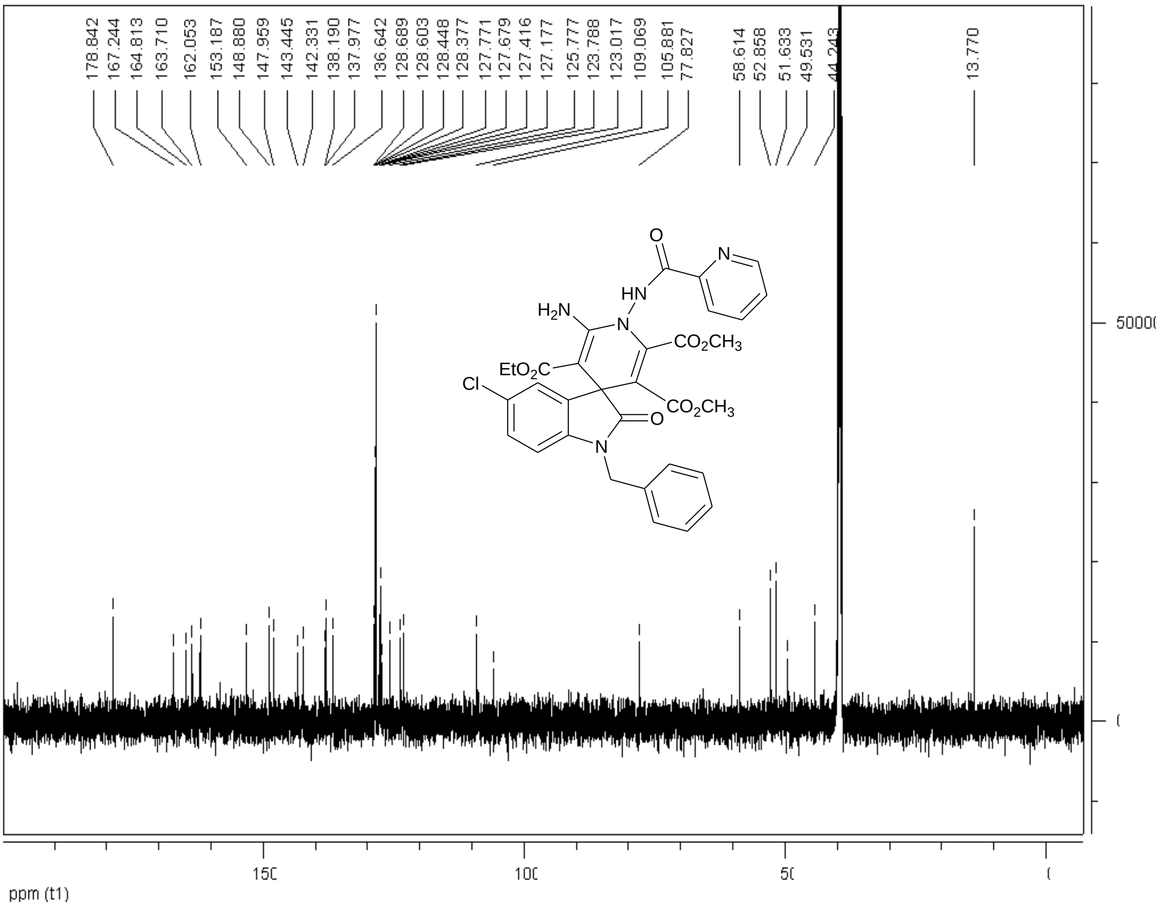

3'-Ethyl 5',6'-dimethyl 2'-amino-1-benzyl-5-methyl-2-oxo-1'-(picolinamido)-1'*H*-spiro [indoline-3,4'-pyridine]-3',5',6'-tricarboxylate1 (1m):

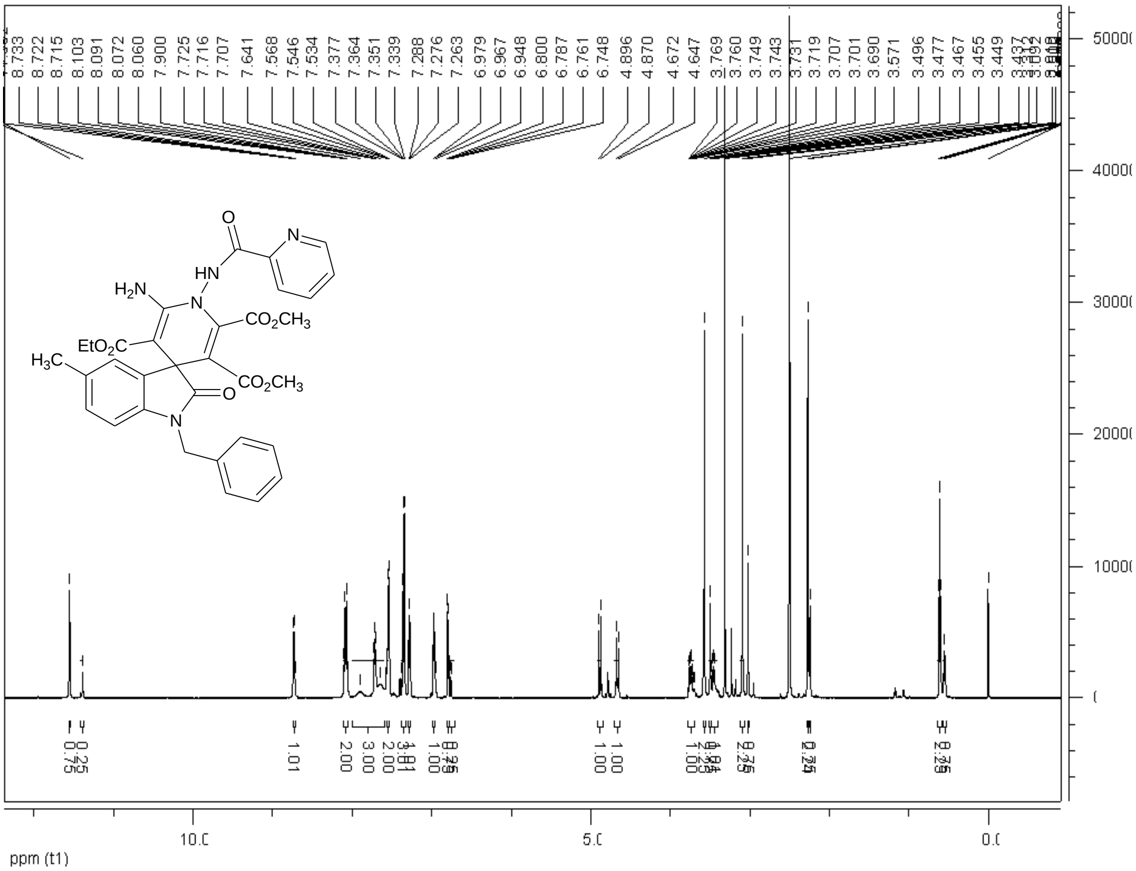

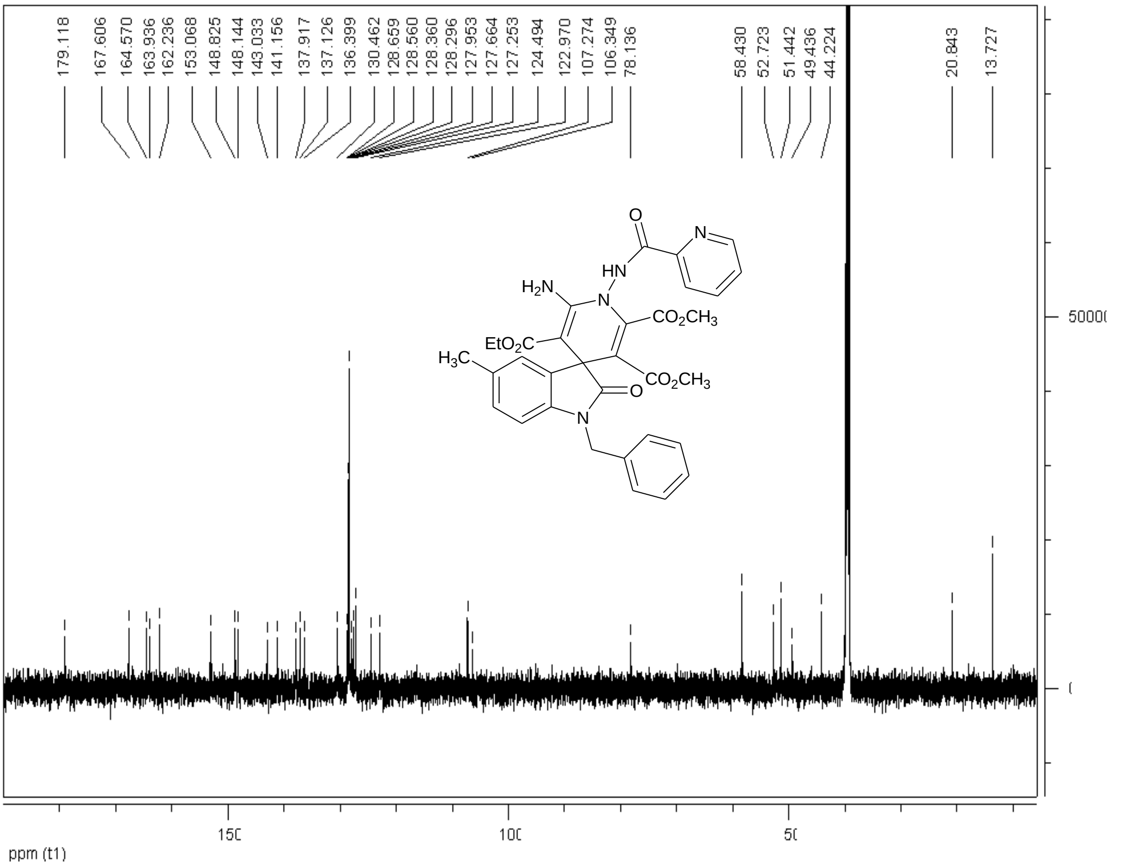

Supplement: File 1 — Experimental details and spectroscopic data of all new compounds. [file Beilstein_J_Org_Chem-10-2671-s001.pdf]
